# Effects of Climate Change and Fisheries Bycatch on Shy Albatross (*Thalassarche cauta*) in Southern Australia

**DOI:** 10.1371/journal.pone.0127006

**Published:** 2015-06-09

**Authors:** Robin B. Thomson, Rachael L. Alderman, Geoffrey N. Tuck, Alistair J. Hobday

**Affiliations:** 1 Oceans and Atmospheres Flagship, CSIRO Marine and Atmospheric Research, GPO Box 1538, Hobart, Tasmania, 7001, Australia; 2 Department of Primary Industries, Parks, Water and Environment, GPO Box 44, Hobart, Tasmania, 7001, Australia; Institut Pluridisciplinaire Hubert Curien, FRANCE

## Abstract

The impacts of climate change on marine species are often compounded by other stressors that make direct attribution and prediction difficult. Shy albatrosses (*Thalassarche cauta*) breeding on Albatross Island, Tasmania, show an unusually restricted foraging range, allowing easier discrimination between the influence of non-climate stressors (fisheries bycatch) and environmental variation. Local environmental conditions (rainfall, air temperature, and sea-surface height, an indicator of upwelling) during the vulnerable chick-rearing stage, have been correlated with breeding success of shy albatrosses. We use an age-, stage- and sex-structured population model to explore potential relationships between local environmental factors and albatross breeding success while accounting for fisheries bycatch by trawl and longline fisheries. The model uses time-series of observed breeding population counts, breeding success, adult and juvenile survival rates and a bycatch mortality observation for trawl fishing to estimate fisheries catchability, environmental influence, natural mortality rate, density dependence, and productivity. Observed at-sea distributions for adult and juvenile birds were coupled with reported fishing effort to estimate vulnerability to incidental bycatch. The inclusion of rainfall, temperature and sea-surface height as explanatory variables for annual chick mortality rate was statistically significant. Global climate models predict little change in future local average rainfall, however, increases are forecast in both temperatures and upwelling, which are predicted to have detrimental and beneficial effects, respectively, on breeding success. The model shows that mitigation of at least 50% of present bycatch is required to offset losses due to future temperature changes, even if upwelling increases substantially. Our results highlight the benefits of using an integrated modeling approach, which uses available demographic as well as environmental data within a single estimation framework, to provide future predictions. Such predictions inform the development of management options in the face of climate change.

## Introduction

Marine and atmospheric environmental factors, such as ocean productivity and air temperature, have been shown to affect the population status of many seabirds including albatrosses [1–4]. Future change in key environmental factors will likely affect seabird populations in both positive and negative ways (e.g. [5–6]). However, the impacts of climate change are often compounded by other stressors. This makes direct attribution of life history changes to environmental stressors, and prediction of their effects, difficult e.g. [7–8]. In the case of seabirds and in particular for long-lived albatrosses, a strong driver of population trends is incidental bycatch in longline and trawl fisheries [9–13] which has been reported to outweigh the effect of environmental drivers e.g. [5]. Any study of the impact of future climate on such populations must, therefore, account for such non-climate interactions. Fortunately, studies of long-lived, central place foragers such as albatrosses, offer an opportunity to differentiate historical environmental effects from non-climate factors and thus more accurately infer the potential impacts of climate change [4–5].

**Fig 1:**
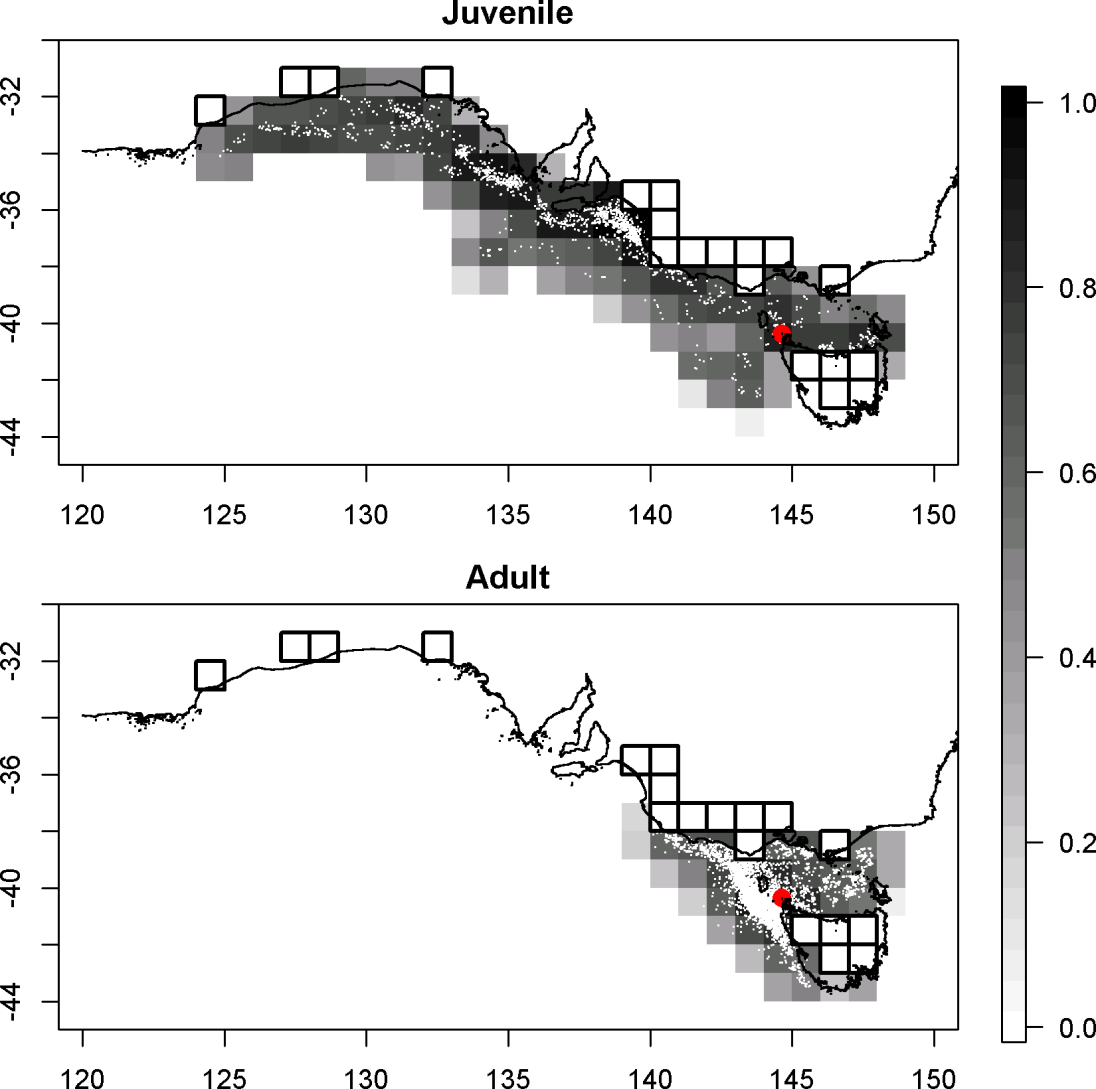
The foraging range for (A) 11 juvenile and (B) 55 adult shy albatrosses from the Albatross Island population. White dots show the locations returned from satellite transmitter (PTT) tracking devices and the colour scale shows percentage utilization calculated using the kernel density method. A 1 degree grid is imposed; black outlines indicate grid cells that have been excluded from the calculation because they substantially cover land. A red dot marks the location of Albatross Island.

Shy albatrosses (*Thalassarche cauta*) are endemic to Australia, where they breed on just three Tasmanian islands: Pedra Branca, Mewstone and Albatross Island. Of these, the Albatross Island population in Bass Strait is the best studied with a 20-year time-series of counts of occupied nests, fledglings and banding data from which adult and juvenile survival rates have been estimated [14] along with sporadic counts from earlier times [15–17]. During the first half of the 19^th^ century, adult shy albatrosses on Albatross Island were heavily harvested for their feathers and eggs [14,18,19], with the population reduced to as few as 400 pairs [20]. Shy albatrosses from the Albatross Island population have an unusually limited foraging range for an albatross, typically restricted to waters between the western boundary of the Great Australian Bight where they forage in waters enriched by the Bonney Upwelling and the eastern limit of Bass Strait (Fig 1) [21–22]. In these regions they come into contact with a limited number of fisheries, for which relatively complete fishing effort data exist with little concern regarding sizeable unknown catches from illegal, unreported and unregulated fisheries. Thus, resolving the historical influence of climate and non-climate drivers (i.e. fishing) may be possible for this species, allowing improved predictions of population trajectories.

Many authors have demonstrated correlation between demographic parameters of seabirds and environmental or climatic measures [2,4,5,23]. Some researchers involved in long-term albatross studies have also used population models (usually matrix population models) to forecast the effect of future changes in the environment on the seabird population size [6,24,25,26]. When the effects of fishing were included in these models they were generally greater than those of the environment [4,5]. Disease has also been identified as an important threat to the stability of albatross populations with virulence linked to environmental variables, in particular to rainfall and temperature [26–28].

These studies typically quantify relationships between environmental variables and demographic parameters (breeding success or survival) and then incorporate the parameter values of any significant relationships found into population models in order to translate the effect of these relationships into a rate of population growth or decline [6,24–26]. Here we present an analysis framework in which all sources of data, i.e. environmental, demographic, and fisheries, are incorporated into an estimation framework. This improves on earlier work in two important ways (i) it incorporates error from all steps in the process, and (ii) it partitions variability due to environmental factors from variability due to other processes such as density dependence and trend in the size of the population [29–30]. Breeding success on Albatross Island, for example, has declined over the past 13 years; both rainfall and breeding population size increased over the same time period and these are significantly correlated. However, this correlation does not account for decreases in breeding success expected to result from density dependent effects as the population recovers from depletion due to egg and feather harvesting. Even if population size were included in a general linear modeling framework alongside rainfall, data available from earlier population counts, before the collection of breeding success information, could not be included. Here we attempt to overcome these problems using an integrated analysis framework that incorporates all sources of error and accounts for demographic, environmental and fisheries processes in the same model [29–30]. We apply an integrated model to shy albatrosses breeding on Albatross Island, Tasmania, Australia, that allows the estimation of environmental factors on breeding success within the model. The resulting parameter estimates were combined with predictions of future rainfall and temperature patterns from climate models to forecast future population sizes for this seabird under a range of future management strategies, including mitigation of incidental catch by fishing operations. The shy albatross (listed as vulnerable under the IUCN criteria [31]) population on Albatross Island is recovering from past heavy anthropogenic impact, is subject to incidental mortality in fishing operations, and, is potentially vulnerable to altered climatic conditions at the breeding site. The model we have developed can provide key management insights into the potential impacts of fisheries and climate change on population conservation status, and can also be applied to similarly impacted species such as other seabird species, turtles, and marine mammals [32].

## Materials and Methods

### Fishing Data

**Fig 2:**
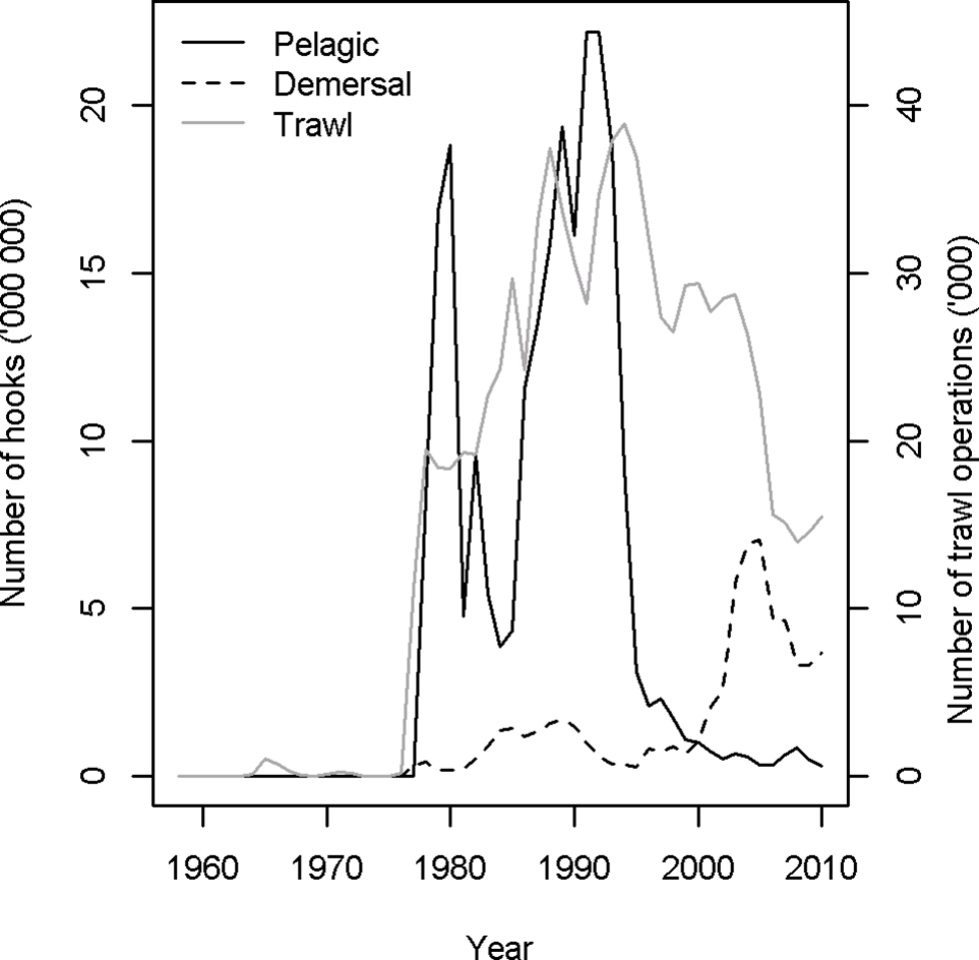
Annual totals for fishing effort in millions of hooks or thousands of trawl operations for pelagic longline, demersal longline, and trawl fishing within the area modelled.

Seabirds are captured during longline fishing operation through seizing baits and subsequently becoming entrapped on hooks [33]. During trawl operations seabirds, including albatrosses, can be either tangled in the net, or dragged under by net sonde or warp cables, particularly during offal discharge [13]. Fishing effort data were obtained for all notable fisheries operating within the core foraging area of the Albatross Island shy albatross population. Of these, trawl and longline vessels are believed to be the main operations that affect seabirds through incidental capture [33–35]. The majority of data were sourced (during 2012) from the Commonwealth logbook database held by the Australian Fisheries Management Authority (AFMA). AFMA data can be requested through the AFMA data manager (see www.afma.gov.au). AFMA data may be released subject to confidentiality and other criteria set out in AFMA’s information disclosure policy. Data were also sourced (during 2012) from a CSIRO held database containing research fishing effort and from the Department of Primary Industries (DPI), Victoria (contact CSIRO, www.csiro.au and www.dpi.vic.gov.au). Fishing effort that overlaps with the shy albatross foraging range is shown in Table 1. We grouped all trawl fisheries into a single trawl super-fleet and divided all line fisheries into pelagic and demersal longline super-fleets (Fig 2). This division reflects the assumption that vessels of a particular super-fleet have the same chance of catching a shy albatross if present. Additional information on the fishing effort used and the underlying fleets is given in S1 Appendix (Supporting Information).

**Table 1:** Fisheries included in the model, showing the super-fleets they have been grouped into and their total effort within the bird foraging area.

*Trawl super-fleet*	*Effort overlapping with birds*
South East Trawl Fishery 1985–2011	276933 operations
Great Australian Bight	82104 operations
Victorian Trawl Fishery–state records (1978–1997)	19984 operations
CSIRO trawl research	3135 operations
Victorian Inshore Trawl Fishery–commonwealth records	956 operations
Victorian Trawl Fishery–state records, fish trawl	*759 operations
Small Pelagic Fishery	754 operations
CSIRO trawl research in AFMA database	180 operations
AFZIS Foreign trawl 1974_1997	80 operations
High Seas South East Trawl	49 operations
Jack Mackerel Trawl	49 operations
AFMA AFZIS Radio Reporting database	49 operations
***Pelagic longline super-fleet***	
Victorian snapper fishery–state records	12.0 million hooks
Tuna Fishery	8.8 million hooks
AFMA AFZIS Radio Reporting database	7.7 million hooks
Southern and Western tuna and billfish fishery	0.6 million hooks
Eastern Tuna & Billfish Fishery	0.07 million hooks
***Demersal longline super-fleet***	
Gillnet, Hook and Trap Fishery (auto-line)	52.9 million hooks
Victorian shark fishery–state records	9.7 million hooks
South East Non-Trawl Fishery *auto-line)	5.2 million hooks
Gillnet, Hook and Trap Fishery (bottom line)	4.0 million hooks
Southern Shark Fishery (bottom line)	1.1 million hooks
South East Non-Trawl Fishery (bottom line)	0.7 million hooks
Southern Shark Hook Fishery (bottom line)	0.12 million hooks
Southern Shark Gillnet Fishery (bottom line)	0.01 million hooks

* 209 days converted to number of trawl shots using the ratio of days to trawls in the Victorian otter trawl fishery.

### Biological data

Annual monitoring of the shy albatross population on Albatross Island was implemented by the Department of Primary Industries, Parks, Water and the Environment (DPIPWE) in 1980. The primary focus of this program is to monitor trends in the number of breeding pairs, annual breeding success and survival and recruitment rates via capture-mark-recapture. Foraging studies have also been undertaken using satellite telemetry and geolocation, with additional information obtained from band returns [14,21,22,36]. More information can be found in S2 Appendix.

#### Tracking data

Satellite tracking data were obtained from 11 post-fledging juveniles and from 55 adults during all phases of the breeding cycle (pre-breeding, incubation and chick-rearing) ([21–22] and DPIPWE unpublished data for 2004 and 2005). These were used to derive utility distributions for the Albatross Island population. Shy albatross tracks can be obtained from the BirdLife International Tracking Ocean Wanderers database *(* www.seabirdtracking.org). These were calculated using a kernel density method based on that described by [37]. The R argosfilter package [38] was used to apply speed and ‘spike’ filters. Linear interpolation was applied when any two fixes were more than 1 and less than 24 hours apart to ensure an even spread of locations before applying the kernel filter. The resulting utility distributions were rasterized into a 1 degree square grid for calculation of overlap with the similarly gridded fisheries effort data.

#### Demographic data

Data on the estimated number of annual breeding pairs, chicks fledged (and therefore breeding success), and adult and juvenile survival rates for the Albatross Island population were sourced from [14] and references therein (Table 2). A previous study [14] used a multi-state capture-mark-recapture (CMR) model implemented in M-SURGE [39] to estimate survival rates. Of the range of model architectures applied by [14], we used estimates from the one that had the most support (lowest quasi-AIC), which allowed a fixed annual adult survival rate value, estimated to be 96.1% (SE 0.45) [14], see S2 Appendix. The number of breeding pairs in 1972 was estimated from a report of the number of occupied nests observed [16]. We assumed that this represented the number of nests containing a chick. However, it might have been the number observed to be in use in the recent past, or the number that contained a chick or an adult, either of which would give a higher count. Our estimate of 12 000 breeding pairs is therefore a possible overestimate. For this reason we tested model sensitivity to a value of 1500 breeding pairs (the lowest number observed during the monitoring period).

**Table 2:** Biological data available from long-term monitoring studies on Albatross Island (taken from [14]).

Year	Breeding pairs^a^	Fledged^c^	Breeding success^e^	Juvenile survival^f^	Return rates^g^
1972–73	2300^b^				
1981–82				0.69	
1982–83				0.70	
1983–84				0.66	
1984–85				0.85	
1985–86				0.72	
1986–87				0.86	
1987–88				0.57	
1988–89	3736 ^c^			0.73	
1989–90	4057 ^d^				
1990–91				0.48	
1991–92					
1992–93				0.63	
1993–94				0.67	
1994–95				0.68	
1995–96				0.29	
1996–97				0.71	
1997–98				0.48	
1998–99	3703	1889	0.51	0.30	
1999–00	3699	1517	0.41	0.30	
2000–01	4326	2033	0.47	0.24	0.96
2001–02	4797	2542	0.53	0.11	1
2002–03	4687	2437	0.52	0.09	0.98
2003–04	5145	2933	0.57		1
2004–05	5640	2707	0.48		1
2005–06	5614	2414	0.43		1
2006–07	5074	1776	0.35		0.9
2007–08	5134	2105	0.41		0.9
2008–09	5212	2241	0.43		0.9
2009–10	5233	2041	0.39		0.95
2010–11			0.38		0.93

Estimated number of breeding pairs, number of chicks fledged at the end of the breeding season, breeding success (number fledged divided by number of pairs), juvenile survival rates to age 1, and annual return rates (proportion of birds that attempt to breed, having made an attempt in the previous season), are shown. Missing data indicates that the information was not collected in that year.

^a^ By chick extrapolation (unless otherwise stated)

^b^ [14] report 1500 occupied nests on 24 January 1973; Alderman (unpublished data) found an average hatching failure of 66%, giving 2300 breeding pairs

^c^ Ground counts

^d^ Area density

^e^ Calculated as number fledged over number of breeding pairs

^f^ From a multi-state capture mark-recapture model [14]. Given the range in age at apparent recruitment (5 to 12, peak at 9 yrs) survival rates are not estimated for cohorts from 2003 onwards and are likely underestimates in the last few years of the time-series presented. This is accounted for in the population model.

^g^ From mark-recapture data (Alderman, unpublished data)

We estimated age at recruitment into the breeding population manually from the mark-recapture dataset by calculating the proportion of individuals recorded as breeding for the first time in each age class. The distribution of ages-at-first breeding is likely to be skewed towards older ages due to the likelihood that the first breeding attempt is not observed for all birds (Table 3). The breeding frequency, or rate at which birds breed following an attempt in the previous season, is likely to be influenced by environmental conditions in the foraging habitat such that the decision to breed will depend in part on the physical body condition that the birds attain during the non-breeding period. Annual breeding frequency was estimated for the 2000 to 2011 breeding seasons (Alderman unpublished data) (Table 2). Outside of those years the average (95%) was used.

**Table 3:** Probability of first breeding at given age (unpublished data) expressed as a percentage.

Age	First breeding (%)
5	1
6	7
7	21
8	39
9	54
10	67
11	78
12	86
13	91
14	94
15	96
16	100

This is likely to be skewed towards older ages because some birds may be missed in their first year of breeding. Insufficient data are available to calculate gender specific values.

#### Bycatch data

An observed ‘shy-type’ albatross capture rate of 36 birds per thousand trawls (31 birds from 856 trawls) was reported in 2006 for the Commonwealth Trawl Sector (CTS) of Australia’s Southern and Eastern Scalefish and Shark Fishery (SESSF) [40] (taken to be in the region between 37°S and 45°S latitude and 135°E and 151°E longitude). According to population estimates for the mid-2000s, the colony on Albatross Island represents approximately 35% of the total number of shy albatrosses (5200 on Albatross Island, 9500 on Mewstone and 170 on Pedra Branca) [14]. Therefore the bycatch rate for the Albatross Island population alone was taken to be 35% of the observed rate, or 13 birds per 1000 trawls. Here we make two assumptions: (1) the observed number of kills represents the total number of shy albatrosses killed and (2) birds from all colonies were caught in proportion to their colony size and none were the essentially morphologically indistinguishable white-capped albatrosses *(Thalassarche steadi)*. These assumptions, if incorrect, would lead first to an underestimate due to unsighted/unreported deaths, and secondly to an overestimate due to the presence of white-capped albatrosses ([41] found 32% of longline caught shy-type birds in Tasmanian waters were white-capped albatrosses). Although much of the longline effort was outside the core foraging areas identified for Albatross Island birds in this study, [40] predominantly recorded visible mortalities (i.e. birds impaled on warp splines or captured in fishing nets), not those associated with cryptic warp strikes [20]. Given these inaccuracies, relatively low weight was given to the bycatch estimate when fitting the model and the sensitivity of the model to this bycatch rate is tested.

### Ethics statement

Ethics approval is given annually by the DPIPWE Animal Ethics Committee and all work was conducted under the DPIPWE Scientific and Threatened Species Permit.

### Environmental variables

Previous work showed that several environmental variables (rainfall, maximum temperature and sea surface height anomaly) recorded during the chick rearing period, were correlated with breeding success of Albatross Island shy albatrosses [20], see S2 Appendix. The observed values for each of the environmental factors included in the model were standardized by subtracting the mean and dividing by the standard deviations calculated across the available time series.

#### Rainfall

**Fig 3:**
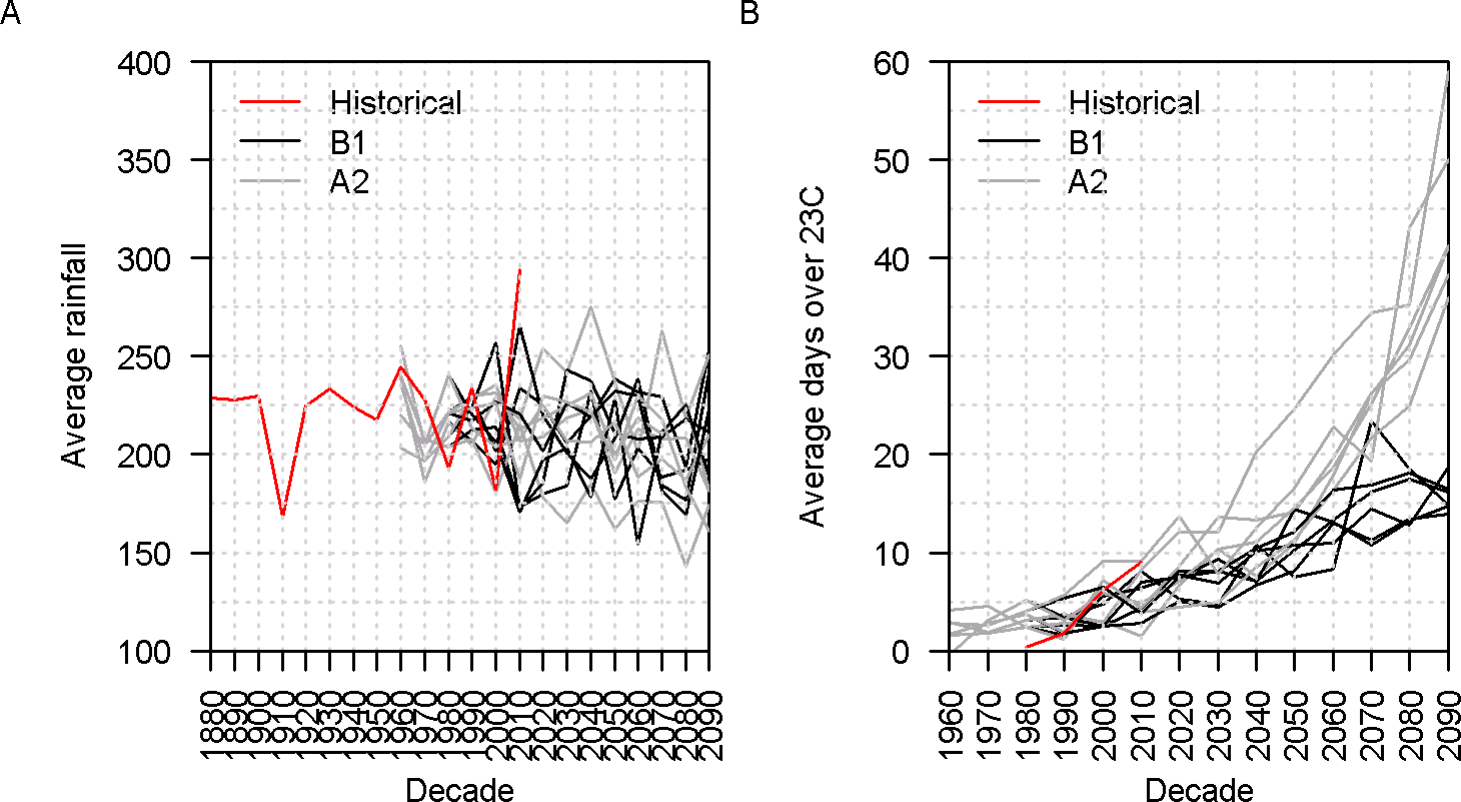
Decadal averages for (A) rainfall and (B) number of days over 23°C at Albatross Island during the chick rearing season for the historical period (red), and hind- and forecasts from climate models using the B1 (black lines) or A2 (grey lines) scenarios. Six variants are available for each of the B1 and A2 scenarios.

Rainfall data are available from 1888 to the present from the Cape Grim (North West Tasmania) weather station approximately 35 km SE of Albatross Island (www.bom.gov.au/climate, station 091011). Days where a reading was not available were allocated the average rainfall for that day of the year calculated over the whole dataset (Fig 3A).

#### Maximum temperature

Maximum daily temperature records are available from the Cape Grim weather station from 1985 (www.bom.gov.au). As reported for a range of other surface nesting seabirds high temperatures affect breeding success through overheating and subsequent mortality [32], and are similarly likely to affect shy albatross chicks [20]. Overall temperature stress was calculated as the number of days with a maximum temperature exceeding 23°C. This threshold was chosen because in exploratory analysis it showed higher correlation with breeding success than any other threshold value and much higher correlation (correlation coefficient of -0.78) than the mean maximum temperature over the chick rearing period (correlation coefficient of -0.44). The decadal average number of days over 23°C is shown in Fig 3B. Decadal averages are used to facilitate comparison with output from global climate models.

#### Sea Surface Height Anomaly (SSHA)

Historical sea surface height anomaly values for each year, averaged over three spatial and temporal scales matching foraging times and areas for shy albatrosses [20] were extracted from a gridded product of MSLA (Maps of Sea Level Anomaly) produced by AVISO based on TOPEX/Poseidon, Jason 1, and ERS-1, ERS-2, Envisat and GFO [42]. This product provides sea level anomalies relative to a seven-year mean from 1993 through 2003. It consists of maps produced every seven days on a 1/3×1/3° Mercator grid, and has been corrected for all geophysical errors.

#### Climate projections

Projected values for future environmental variables were obtained from several sources. Daily rainfall and air temperature time series were sourced from the Climate Futures Tasmania data repository (https://dl.tpac.org.au/tpacportal/#category=17) for the pixel overlying the location of Cape Grim. Six different global climate models for each of two future IPCC emission scenarios (A2 “high” and B1 “low”) have been dynamically downscaled to produce fine-resolution (approximately 10 km × 10 km) projections of key climate variables for Tasmania over the 21^st^ century [43]. Model derived total rainfall (decadal averages) during the chick rearing period is shown in Fig 3A. The models also back calculate rainfall to 1960 so that an overlap exists with observations for the 1960 to 2010 period. During this period the average rainfall during the chick rearing period from the climate models is 61.5 mm higher than that of the observations; consequently the forecasts were adjusted downwards by 61.5 mm [44]. Decadal averages for the observed and model-derived rainfall totals during the chick rearing period are shown in Fig 3.

Future average daily temperatures from the climate models had to be converted to maximum daily temperatures for use in the population model. This was achieved by regressing average temperature from all models pooled, against observed maximum daily temperature and applying this correction to all temperature values from the climate models. The regression was achieved by calculating the percentiles (5^th^, 6^th^ ….94^th^, 95^th^) for each dataset for the overlapping period (the first four months of each year 1986–2012) and regressing those, rather than attempting to match the observed maximum with the modelled average for particular days of the year. Decadal averages for the resulting estimate of the number of days over 23°C during the chick rearing period are shown in Fig 3.

Future sea surface height data, averaged for the same spatial and temporal regions as for historical data, were obtained from a dynamically downscaled GCM projection based on the CSIRO Mk3.5 A2 scenario (see [45] for the years 2063–73). The GCM projection is dynamically downscaled using the Ocean Forecasting Australia Model (OFAM) [46]. OFAM is eddy resolving in the Australian region (0.11 of longitude and latitude), which allows representation of mesoscale features that influence sea surface height in the region.

The OFAM model suggests that there will be more negative SSHA in the future, but as there is considerable drift in these model forecasts and the baseline conditions for SSH are unclear, these quantitative data were not included. Instead, we considered the effect of stronger negative SSHA values (i.e. increased future eddy-related upwelling) into the future based on increases from observed values of up to 300%.

**Fig 4:**
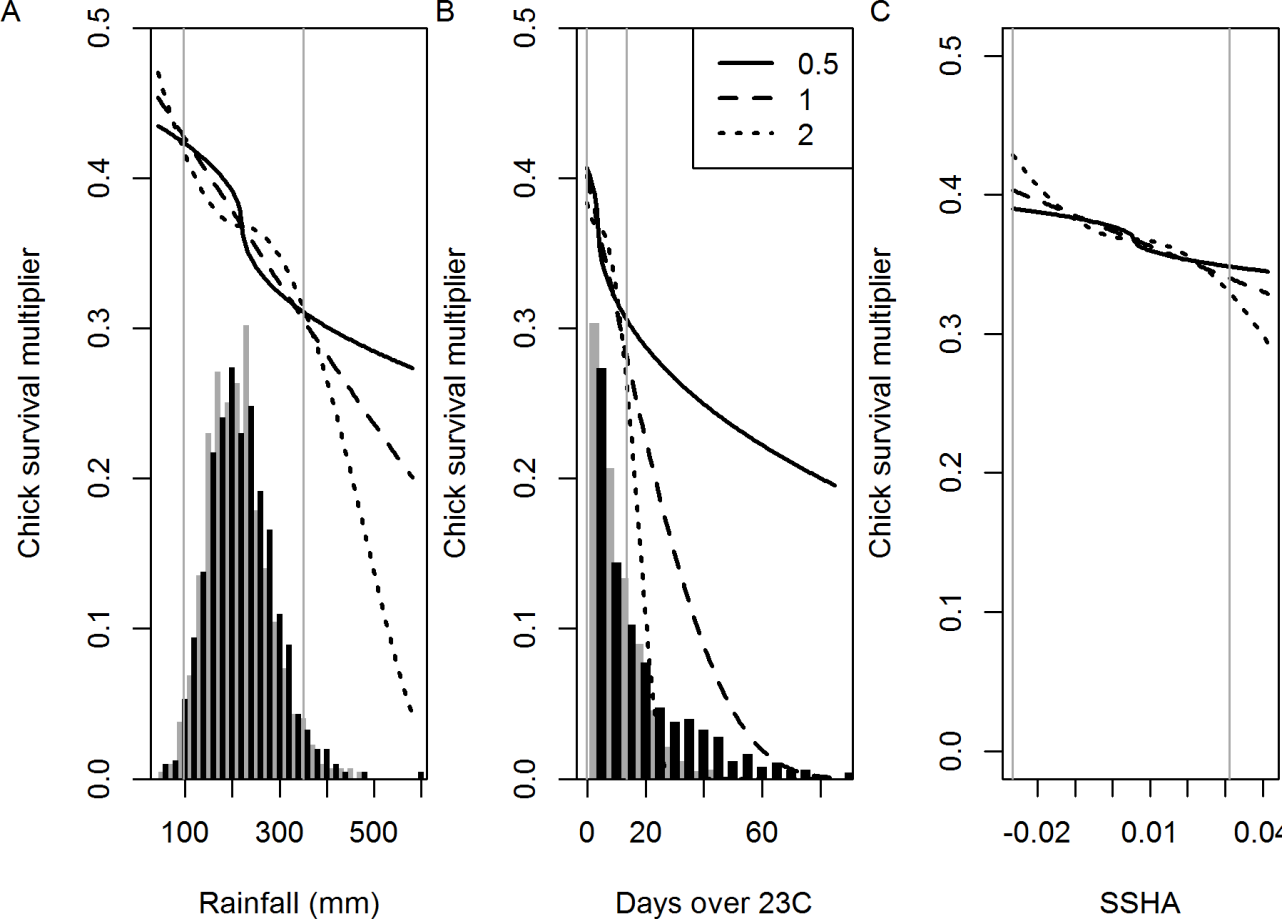
The estimated relationships between a multiplier on chick survival and (A) rainfall, (B) number of days with a maximum temperature over 23°C, and (C) SSHA. The histograms reflect frequency distribution of rainfall and temperature from the B1 (black bars) and A2 (grey bars) climate models. Vertical lines show the range of the historical measurements. Note that SSHA forecasts are not available, but an observation for 2011 (the model ends with 2011) was used. The functional forms shown use different values for a power parameter *b* which is chosen to be 0.5, 1 or 2.

Since the 1970s, Tasmania has experienced a general trend of reduced annual average rainfall, and greater year-to-year variability in rainfall [43]. The modelling of changes to rainfall under the A2 (high) emissions scenario suggests a change in the total annual rainfall for Tasmania of less than 100 mm by the end of the 21^st^ century. This minor state-wide change masks significant changes in regional and seasonal rainfall patterns. Both emissions scenarios project significant regional differences in rainfall, with an increase in rainfall projected for the west coast of Tasmania. During summer and autumn, the west coast, which is closest to Albatross Island, is anticipated to experience reduced rainfall while during winter and spring, rainfall in the west is expected to increase [43]. For both the B1 and A2 scenarios, only 12% of the predicted future annual rainfall values (at Cape Grim during the four months of the chick rearing period) exceed the historical range (Fig 4).

Throughout the first half of the 20^th^ century, Tasmania experienced a stable mean land temperature. Since 1950, mean land surface temperatures in Tasmania have increased by an average of 0.10°C per decade, which is a slower rate of increase than for mainland Australia (0.16°C per decade) [43]. Projections for changes over the 21^st^ century indicate that average annual temperatures across Tasmania will increase between 1.6°C- 2.9°C by 2100. Increases in temperature for Tasmania are less than that projected for global average temperatures and for the Australian mainland, mainly due to the moderating effect of the Southern Ocean. Increases in average land surface temperatures are matched by increases in both maximum and minimum temperatures, with daily minimum temperatures projected to increase slightly more than daily maximum temperatures. Notwithstanding existing variations in temperature based on changes in elevation, the average annual increase is anticipated to be relatively uniform across Tasmania. There are, however, seasonal spatial differences in projected temperature rises, with the west coast of Tasmania experiencing greater increases during summer [43]. Of the predicted future temperature “threshold” counts (days over 23°C at Cape Grim during the four months of the chick rearing period) 23% of those for the B1 scenario and 38% of the A2 scenario exceed the historical range (Fig 4).

### Seabird Population Model

The seabird population dynamics model is based on the integrated modeling framework introduced by [11] and [47] but a number of changes have been made. Estimation is achieved using likelihood instead of least squares; the yearly time step for the age and stage structured model has been changed to monthly, allowing for more accurate estimation of bird-fishery overlap; environmental covariates are allowed to alter chick mortality and thereby, breeding success; a breeding season of less than 12 months is allowed; male and female birds are modelled separately, allowing differential fishing mortality, observed bycatch rates are used to condition the model; and the population is not considered to be pristine at the commencement of significant fishing activities.

At-sea bird distribution data collected using satellite trackers are combined with reported spatial fishing effort data, to calculate bird-fishery overlap and consequent vulnerability to fisheries bycatch. Intensive feeding is required for the survival of an albatross chick [48] therefore we assume that the death of either parent leads to breeding failure (the death of the chick). The full specifications for the population model are given in S3 Appendix (Supporting Information).

At least some life-history traits must be pliable and able to respond to changes in population size so that populations have protection from extinction and cannot grow beyond the bounds fixed by limiting factors (such as food resources or space) for extended periods [49]. This density-dependent compensation also allows populations to stabilize, albeit at lower population sizes, when moderate levels of increased mortality (e.g. due to fisheries bycatch) occur. [47] found that the wandering albatross (*Diomedea exulans*) colony on Possession Island showed density-dependent compensation in both juvenile survival rates and breeding success [50–51], the ecological basis for which might be a decrease in the intra-specific competition for resources amongst juveniles and adults foraging for their chicks [52]. The model developed here also allows both forms of density dependence, but initial investigations showed that the data supported density dependent chick survival and not juvenile survival. Information on the initialization of the model, which commences in 1942, and the use of a parameter to describe harvesting pressure prior to that year, is given in S3 Appendix (Supporting Information).

The model year runs from the start of the breeding season, 1 October, to 30 September the following year; for example, 1994 refers to the year that starts 1 October 1994. The breeding season finishes at the end of May, when all surviving chicks have fledged. The model is applied to an area bounded by latitudes 45°S and 30°S and by longitudes 123°E and 151°E, which encompasses the unusually restricted foraging range for Albatross Island birds (Fig 1).

The pre-exploitation size of the Albatross Island population was estimated at 11 110 pairs from the extent of old guano deposits coupled with the current known nesting density for these birds [14]. We used this estimate to set the carrying capacity for the population at 12 000 pairs. The estimates of juvenile survival derived from an M-SURGE [39] mark-recapture model in which the assumption was made that juvenile birds had the adult mortality rate from age 2 to 5 (the earliest age at first breeding). Because birds are not re-sighted until their first breeding attempt, it is difficult for a mark-recapture model to tease apart annual mortality rates for juvenile birds for each of the years they are at sea. However, our model applies annual fishing mortality rates based on the annual distribution and intensity of fishing pressure overlapping with the juvenile foraging distribution so that the juvenile survival rate differs each year. In order to condition the model on the juvenile survival rates from the mark-recapture study we adjusted the estimates of survival during the first juvenile year to survival to age 5 by adding 3 additional years of adult mortality. This figure is then compared with the model estimated survival to age 5.

The parameters of the model are: adult natural mortality rate, density dependence on juveniles and separately on chicks, breeding success in the pristine population (a measure of productivity), and catchability parameters for the trawl and pelagic longline fisheries. In addition, we estimate parameters that relate environmental variables to chick mortality and consequently breeding success.

We use the population model to quantify the impact of each environmental factor on breeding success by allowing chick mortality in a given year to be a function of the environmental factors. Suppressing subscripts for year (for clarity), chick mortality $M_{y}^{0}$ (which is calculated by the model and is a density dependent function of the size of the breeding population, see S1 Appendix in the Supporting Information) is given by

$$M_{y}^{0}={\bar{M}}_{y}^{0}\;\sum_{i\in I}f\left(x_{i}\right)$$

where ${\bar{M}}_{y}^{0}$ is the instantaneous annual rate of chick mortality (if environmental factors were at their mean levels); and *f*(*x*_*i*_) is a functional relationship for environmental covariate *x*_*i*_ (of the set of factors *I* used by the model). A flexible, exponential, functional form was used

$$f\left(x_{i}\right)=\text{exp}\left(\theta_{i}\;x_{i}{}^{b}\right)$$ (1)

where *θ*_*i*_ are estimated parameters and *b* is fixed at 0.5, 1 or 2, giving differing behaviours outside of the range of *x*_*i*_ values observed during the historical period.

Although these functional forms can give quite different relationships, the model estimated values that gave very similar results over the range of the observed data (Fig 4). However, each gives quite different forecasts for higher future values for the environmental factors.

The time series data for each environmental covariate (*x*) was standardized by subtracting the mean and dividing by the standard deviation for the time series. A value of zero (*x* = 0) thus returns *f*(*x*_*i*_) = 1, so that ${\bar{M}}_{y}^{0}$ represents the chick mortality rate when all environmental factors are at their mean values.

Shy albatross chicks on Albatross Island have been observed to suffer from a disease, probably avian pox virus [20]. It has only been observed in chicks and is most prevalent in one sub-colony that represents approximately 12% of the total island population. Particularly bad outbreaks in 1999 and 2006 influenced breeding success noticeably, but the effect has not been severe in other years. Breeding success data for the affected years were excluded from the dataset, but other than this disease was not considered in the model.

## Results

### Fits to observations

The model using *b* = 1 (the power parameter for the relationship that relates environmental variables to chick mortality in Eq 1) was used to examine the effect of including or excluding climate variables and is examined in greatest detail below. If not otherwise specified, “the model” refers to the version using *b* = 1. The models that used *b* values of 0.5 or 2 gave poorer fits to the observed data (Table 4).

**Table 4:** Negative log likelihoods (-lnL), estimated adult natural mortality rates (*M*) and productivity (the breeding success rate for the pristine stock) with standard errors (SE) for the models that use each of the three alternative values for *b*, the power parameter for the relationship that relates environmental variables to chick mortality.

*b*	-lnL	*M* (SE)	Productivity
0.5	255.3	0.039 (0.003)	0.43 (0.04)
1	251.7	0.041 (0.004)	0.42 (0.03)
2	252.8	0.040 (0.005)	0.41 (0.07)

**Fig 5:**
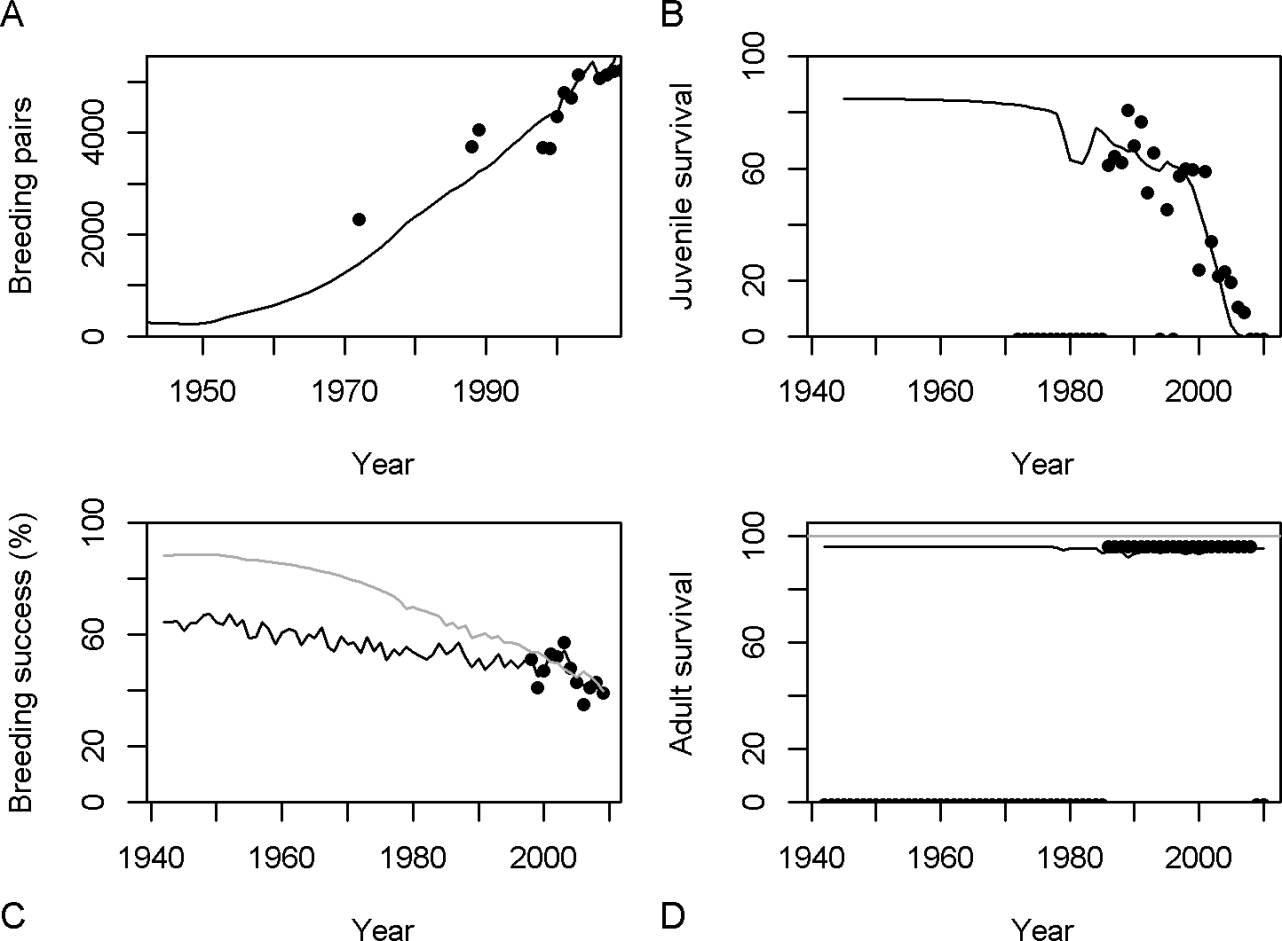
Observed (circles) and expected (lines) annual time series of (A) the number of breeding pairs in the Albatross island population; (B) apparent juvenile survival rate to 5 years old (note that apparent survival for recent years is low because young birds have not all had time to mature); (C) adult survival rate (the grey line marks the maximum value possible: 100%); and (D) annual breeding success (the grey line shows the expected breeding success for the model that ignores environmental factors).

The counts of breeding adults are reasonably well matched and the model achieves a good fit to the juvenile survival rates (Fig 5). Note that the apparent sharp drop in recent survival (in both the observed and model expected values) is an artifact resulting from incomplete recruitment to the breeding population of younger cohorts (Fig 5B), and thus does not necessarily reflect the true juvenile survival rates for those cohorts.

The model estimates an average adult annual survival rate of 94.1% (Fig 5), compared with 96.1% (SE 0.45) from the multi-state CMR model. A weakness of the Albatross Island mark-recapture dataset is that it contains spatial and temporal heterogeneity in search effort and therefore in probability of re-sighting individuals. [14] dealt with this by recognizing two recapture states. However, additional subdivisions might have given more realistic results but would have been difficult to identify and would have over-parameterised their CMR model.

The observed breeding success is matched very well, especially when environmental factors are taken into account (Fig 5). Chick mortality rates, and consequently breeding success, drop from over 60% in the 1940s when the population was low due to harvesting for feathers, to the roughly 40% observed in recent years. While there has been an average increase in the number of days over 23°C and increased rainfall during incubation since 2010 (Fig 3) the primary cause of this reduction has been density dependence, due to which estimated chick survival (independent of adult survival, and before applying environmental influences) dropped from 69% y^-1^ to 50% y^-1^. The observed bycatch rate of 13 birds of ‘shy-type’ per thousand trawls [40] was matched by an estimate of 8 birds per thousand trawls. Similar results were obtained for the two models using *b* = 0.5 and *b* = 2 (not shown). All models give much the same chick survival rates over the range of the historical values of the environmental factors (Fig 4).

### Parameter values

The best fit to the data is achieved with an instantaneous adult natural mortality rate of 0.041 (SE 0.004, Table 4), corresponding to a survivorship of 96% per annum (note that with additional fishing mortality, the average model estimate of adult survival over 1986–2008 is 93%). Over the full time period, similar incidental bycatch is attributed to the trawl fishery and to pelagic longlining (each taking roughly 6 000 birds over the modelled period, which is 1964 to 2010). Greater overlap between the birds and the trawl fishery in recent years leads to the trawl fishery taking 76%, pelagic longline 17% and demersal longline 8% of the incidental bycatch in the most recent five years (2006–2010). Juvenile and chick survival (and therefore breeding success) are estimated to have a density dependent component.

The estimated parameter values showed little change when the number of observed breeding pairs in 1972 was reduced from 2300 to 1500 (not shown). Halving or doubling the single bycatch rate observation for the trawl fishery has little influence on the estimated environmental parameters, with the greatest change being a 6% increase in the slope of the rainfall relationship when the bycatch observation is halved. However, the catchability parameter for the trawl fishery does change, resulting in total catches over the modelled time period of some 3 400, 5 600 and 6 100 birds for the cases where the bycatch observation was halved, unmodified, and doubled, respectively. The model balances this change by manipulating the breeding success of the pristine population (and therefore the extent of density dependent compensation that is allowed) (0.43 in the half, 0.42 in the unmodified and 0.41 in the double case, standard errors are shown in Table 4.).

### Effect of environmental factors

**Fig 6:**
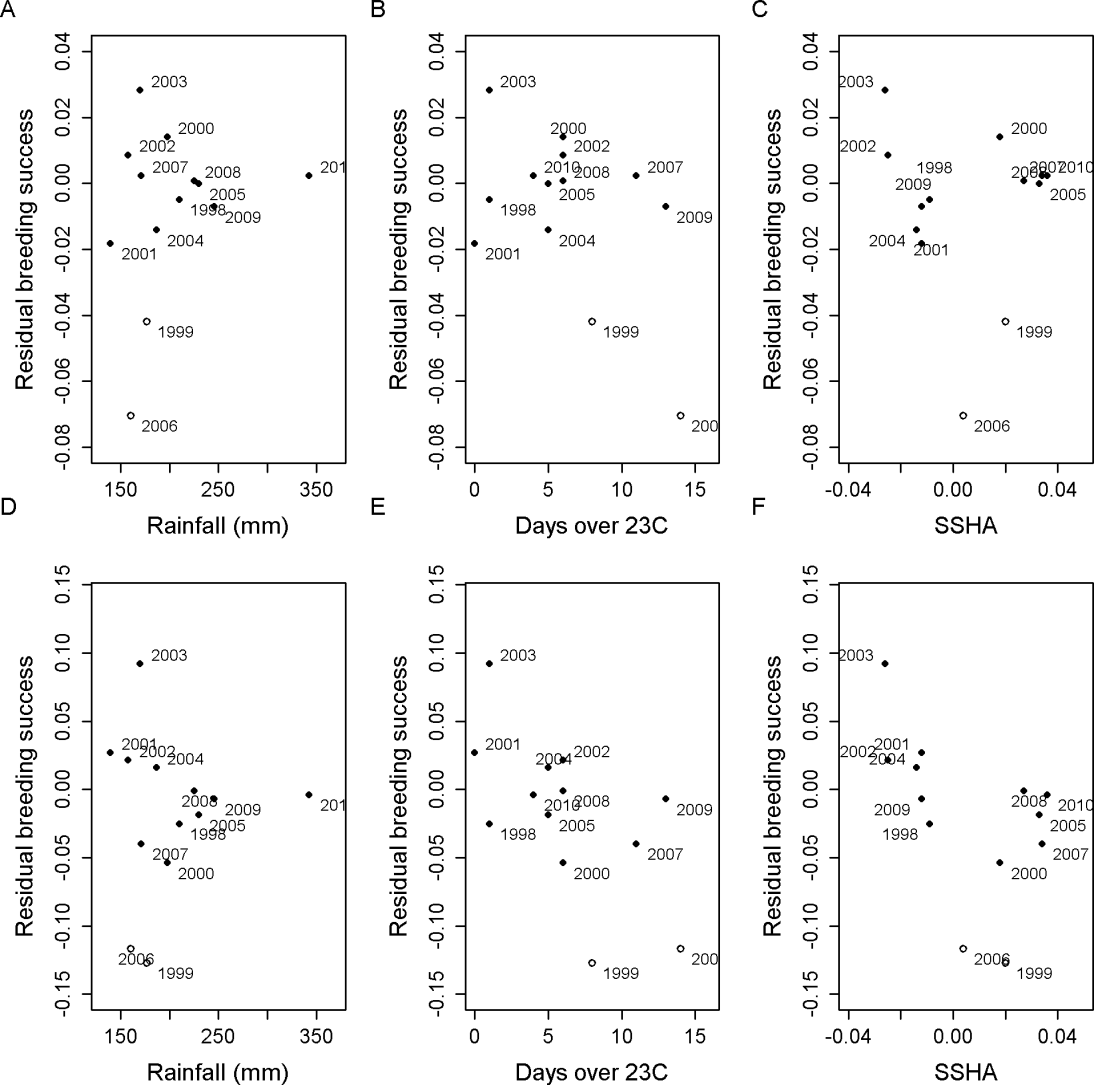
Annual observed minus expected breeding success plotted against the standardized environmental covariate values: (A) rainfall (R), (B) number of days over 23°C (T), and (C) SSHA (S) for the model that allows environmental factors to influence chick mortality (‘RTS’); and (D,E,F) the model that ignores environmental factors (‘xxx’). The values for 1999 and 2006 are shown as open circles because these were not used in the model, having been years of notable pox infestation.

The addition of all three environmental factors significantly improved the fit of the model to the data (Table 5), both singly and in combination. The inclusion of any one, or any combination of, the environmental data time series with their associated parameters was statistically significant (Table 5). The likelihood ratio tests (Table 5) evaluate the inclusion of environmental time series relative to the model that has none (`xxx’). Comparisons between models containing a single environmental series (Rxx, xTx, xxS) and those containing that series and just one other (xTS, RTx, RxS), were all statistically significant (p<0.005) indicating no notable co-linearity between environmental series. Positive values were estimated for each environmental slope parameter, indicating that breeding success is detrimentally affected by greater rainfall, more days with maximum temperatures over 23°C, and downwelling (higher SSHA). This is in accord with the significant relationships found using a GLM applied to the observed breeding success and covariate data by [20]. There is no apparent pattern in the residuals after the effect of the environmental factors has been taken into account (Fig 6A–6C, correlation coefficients are -2%, -5% and -1% when 1999 and 2006 are ignored) in contrast with the noticeable negative trends for the model that does not use environmental factors (Fig 6D–6E, correlation coefficients are -27%, -46% and -64% when 1999 and 2006 are ignored).

**Table 5:** Negative log-likelihood values (-lnL) for each of the models considered, the improvement over the null model (xxx) is shown (2*Diff) along with the significance (p) of the inclusion of n additional parameters calculated using the likelihood ratio test.

Model^1^	-lnL	2*Diff	n	p	AIC
RTS	251.7	81.1	3	0.00	512.5
xTS	258.6	67.4	2	0.00	525.2
RTx	261.3	62.0	2	0.00	530.6
RxS	264.8	53.0	2	0.00	537.6
xxS	268.8	46.9	1	0.00	544.6
xTx	278.3	27.9	1	0.00	563.6
Rxx	280.9	22.7	1	0.00	568.9
xxx	292.3				590.5

^1^ RTS indicates that rainfall (R), temperature (T), and SSHA (S) were included; an x indicates exclusion of the variable. Thus xTS indicates that rainfall was excluded, but temperature and SSHA were included.

Although a linear relationship is apparent in the breeding success residuals for rainfall (Fig 6), a simple linear regression applied to these figures is not significant (p = 0.2). However, when the breeding success observations for the two years most heavily affected by disease (1999 and 2006) are excluded, the correlation becomes significant. These two values were excluded from the model fitting procedure.

### Future climate change

The environment-related modifier applied to chick survival rates varies greatly depending on the choice of *b* (Fig 4). A value of *b* = 0.5 gives less modification at extremes of the environmental range compared with a value of *b* = 2, and the *b* = 1 model falls in the middle.

**Fig 7:**
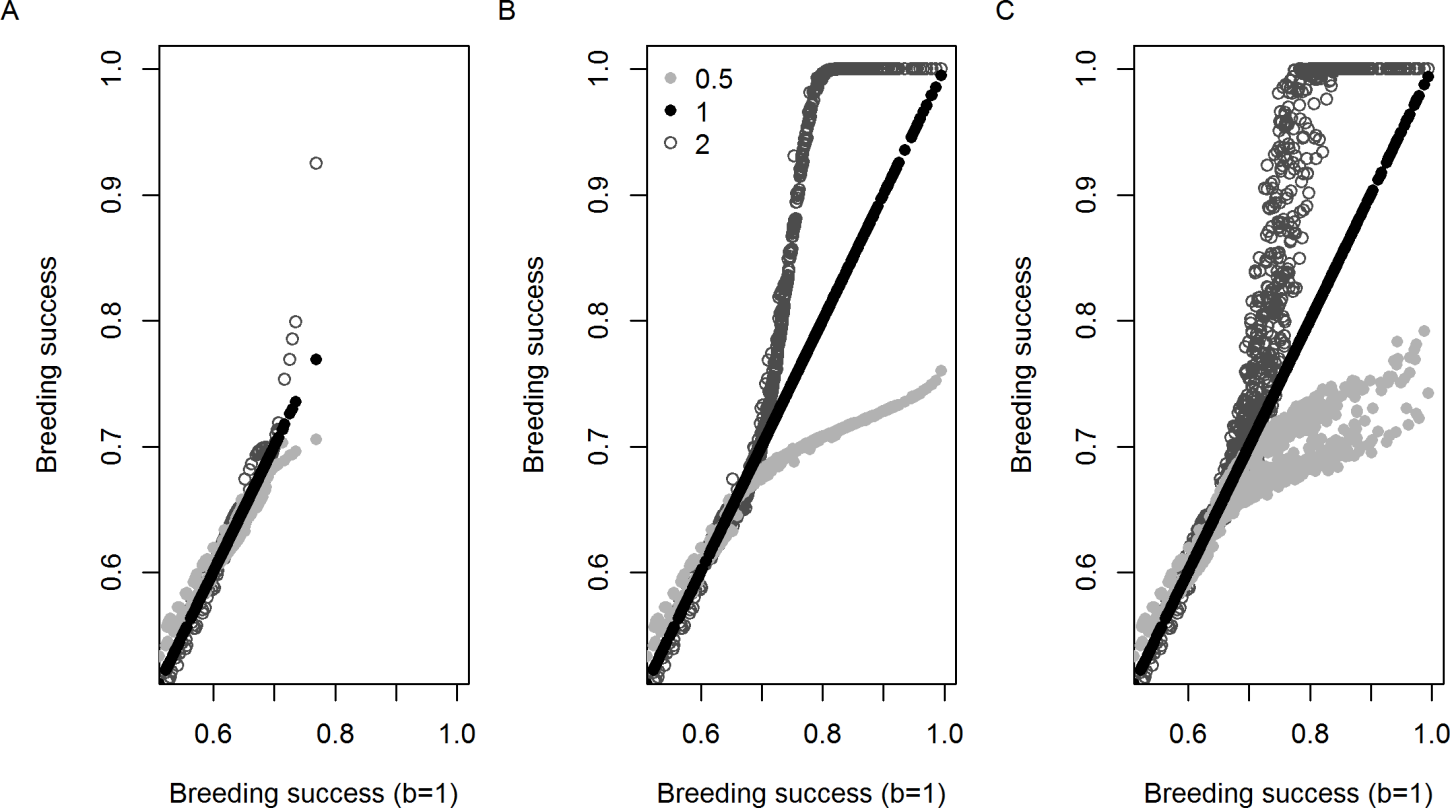
For every year and all 12 future climate scenarios (6 models each using a B1 or an A2 scenario), the estimated breeding success from the three models that used *b* values of 0.5, 1 or 2, are plotted against those from the *b* = 1 model. Results are shown for future projections that (A) use future rainfall but keep future temperature at its historical mean, (B) keep rainfall at its historical mean but use future temperature, (C) use both future rainfall and temperature.

**Fig 8:**
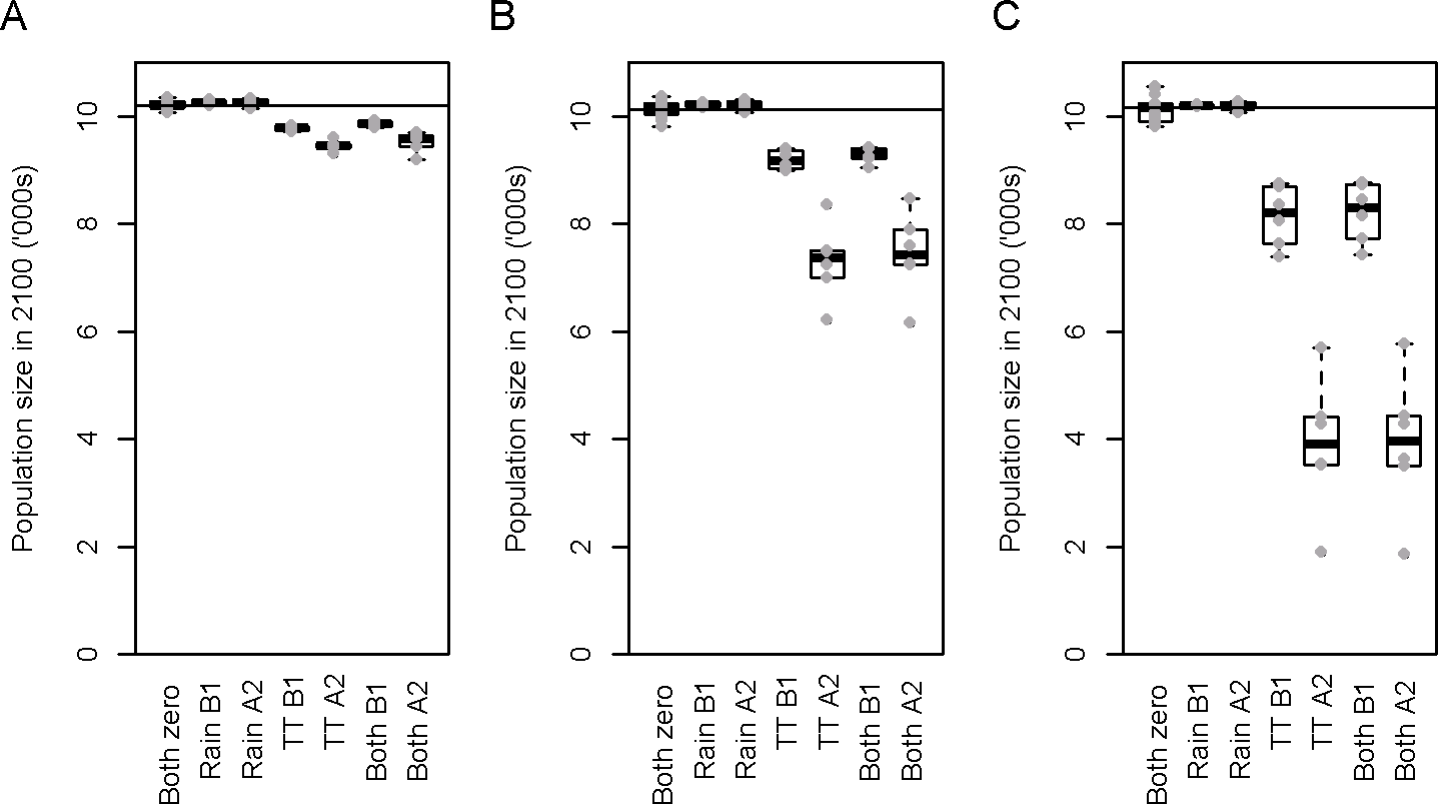
Projected number of shy albatross breeding pairs on Albatross Island in year 2100 if rainfall (“Rain”) and temperature (“TT”) are drawn from a normal distribution with historical mean and variance (“Both zero”) or if either or both are taken from the six B1 and A2 climate projections. Standard box and whisker plots are shown with the actual projected population sizes overlaid (grey circles). Horizontal black lines show the median population size in the climate neutral scenario. Results are shown for the models that use (A) *b* = 0.5; (B) *b* = 1; and (C) *b* = 2.

Future rainfall shows effectively no trend so that values vary erratically from year to year and do not translate into an overall change in population size (Figs 7 and 8). In contrast, future temperatures show a strong increasing trend resulting in greater depression of breeding success (Fig 4) and consequent lower population sizes which in turn, through the density dependent mechanism, give rise to higher breeding success values (Fig 7). Because rainfall varies erratically, instead of smoothly, over time, density dependence cannot act to offset its effect so that the addition of future rainfall along with future temperatures adds greater variation to the estimates of future breeding success as compared with the model that uses future temperature alone (Figs 7 and 8).

As expected, the *b* = 0.5 model shows least spread in the estimates of number of breeding pairs in 2100, and the *b* = 2 model shows the most (Fig 8). Future rainfall caused least change, resulting in almost negligible increases in future population size compared with large decreases for estimated future temperatures. As expected, the hotter A2 estimates result in lower population size than the B1 models. When climate model estimates for rainfall and temperature are combined, median future albatross population sizes of 82–97% (of the median of the climate neutral scenario) result for the B1 scenario, and 39–95% for the A2 scenario (Fig 8). Under the most extreme case, namely the A2 scenario with *b* = 2, the population is reduced to 1 865 pairs (16% of the pristine size) in 2100.

**Fig 9:**
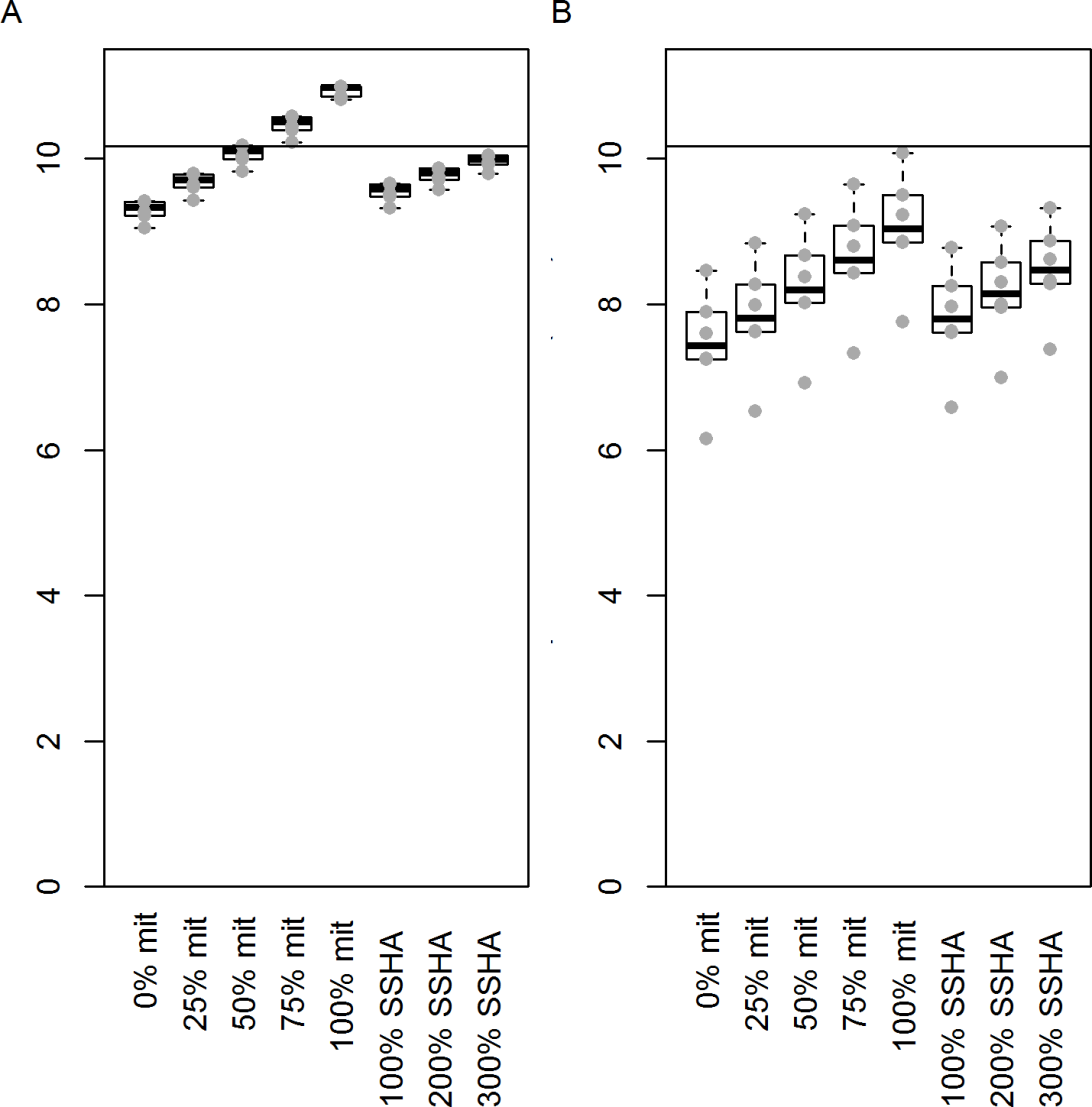
Projected number of shy albatross breeding pairs on Albatross Island in year 2100 based on rainfall and temperature time series from the six (A) B1 or (B) A2 climate projections. Incidental capture by the trawl fishery is assumed to remain the same (i.e. no mitigation: “0% mit”) or to be stopped entirely (100% mitigation: “100% mit”) and values in between. Alternatively, future SSHA values are assumed to be between 100% (“100% SSHA”) and 300% (“300% SSHA”) greater than their historic mean. Box and whisker plots are shown with the projected population sizes from which they are constructed, overlaid (grey circles). These are shown because box and whisker plots based on such a small number of data points can be misleading. Horizontal black lines show the median population size in the climate neutral scenario.

Mitigation of incidental bycatch from trawl fisheries must exceed 50% of the rate estimated by the model (for the years to 2010) in order to offset the effect of climate change, with two of the B1 futures and none of the A2 futures showing higher population size in 2100 in the absence of mitigation (Fig 9). It is not possible to translate the climate model predictions of strengthening of upwelling within the foraging range of the shy albatrosses into SSHA values, however, it is clear that the increase in food production resulting from greater future upwelling may help to offset population size reduction due to future temperatures (Fig 9). An increase of 300% in upwelling seems very large in comparison with historical values, but is insufficient to fully offset other climate-related losses.

## Discussion

Predicting the range of future population trajectories for species is critical in evaluating potential management actions, as is resolving the contribution of different stressors on populations. In many regions, management of threatened and protected species is a high priority, with climate change potentially compounding existing problems. Here we have shown that both climate and fisheries impact the population status of shy albatrosses, and that the situation may become worse under a changing climate.

To date, few studies have combined the impacts on seabirds as a result of climate and fisheries into a single fully parameterized population model [4]. [24] and [53] estimate the relationships between environmental factors and life history characteristics and then introduce these quantified relationships into matrix population models [54] to investigate their impact on a population. [55] allow climate to influence transition probabilities within a stochastic matrix population model initialized using observed population age distribution and size observed in 1962. Demographic parameters are also usually estimated outside of the population model using mark-recapture models such as MARK [56] and M-SURGE [39] and the resulting values are used without error [25,26]. Few, if any, parameter values are estimated and these are conditioned on a single time series of counts e.g. [57]. To the best of our knowledge, our study is the first to have incorporated the estimation of climate impacts into a population model that also estimates fisheries bycatch. In addition, our population model does not use fixed survival rates, and breeding success values from mark-recapture models but treats these as observations with inherent observation error, estimating a range of parameter values conditioned on population counts as well as survival and breeding success estimates. At-sea observations of seabird bycatch are also not used as known values but are compared with estimates. We have constructed a truly integrative framework from which to make predictions and assess management adaptation options for seabirds. This framework is also adaptable for studies of other species, including seabirds, turtles and marine mammals.

The accuracy of the predictions made here are a function of the accuracy of parameter estimation, both in the population dynamics component of the model and in the quantification of the relationships with fishing and with climate variables. First, the observed patterns in the demographic time series are, on the whole, well estimated, giving confidence in the model’s estimated parameter values. Second, the model achieves a good match to the single observation of bycatch rate from a trawl fishery, but attributes virtually all bycatch mortality to that super-fleet and none to the pelagic longline fishery, which seems unrealistic. No observed bycatch rate is available for the pelagic longline fishery, which allows the model the flexibility to greatly alter the amount of mortality attributed to the longline fishery. The model estimates that the trawl and pelagic longline fleets have similar catchabilities, with higher trawl than pelagic longline mortality during 2001 to 2010. This agrees with observations that incidental mortality in the trawl fishery has been high, potentially higher than that in the pelagic longline fishery ([1], Alderman, unpublished data), which is known to have caught a substantial number of albatrosses [33]. In the absence of an observed bycatch rate from the pelagic longline fishery for this population, the model is reliant on comparing the pattern in fishing effort over time with the population response in order to evaluate to what extent each fishery has contributed to bycatch mortality [11]. In the absence of strong differences in the effort time series for the two super-fleets, the data are not informative regarding the split of bycatch between fisheries. In addition, the model estimate of the extent of bycatch mortality in the trawl fishery is sensitive to the value of a single observation of bycatch, which is likely to be poorly estimated. While this parameter uncertainty is not influential on the climate forecast shown here or even on the fisheries mitigation options discussed below, it does mean that the effects of fishing by the trawl and line sectors cannot be reliably disentangled so that the conclusions drawn here relate to fishing in general, not to the trawl sector alone.

Third, the quantification of the relationship between climate factors and breeding success are well estimated over the range of the historical data, but (as for any model) extrapolation outside of this range is speculative and is not informed by data. We explored this by fitting three alternative functional forms to the data, two of which gave noticeably different answers. Therefore attention should be focused on the general trends shown in our results e.g. the population size is low under future rainfall and climate forecasts, rather than on absolute changes. We used a range of functional forms that gave a similar relationship between the climate variables and the breeding success residuals over the historical period, but quite different forecasts; other functional forms could also have been used. As climate change progresses, with continued monitoring of the shy albatross population as well as of similar populations around the world, we will observe the responses of populations to more extreme environmental conditions than those encountered historically, and will therefore be able to reduce the range of candidate functional forms. Specifically, seabird species have been shown to demonstrate plasticity in their ability to respond to changing climate [3] e.g. by changing breeding phenology. The diverse diet of shy albatrosses and their year-round residency within a comparatively restricted geographic range suggests this species has the capacity to respond to changes. Similarly, plasticity in prey species, competing predator species, and disease, could influence the response of this species to future climatic conditions. This highlights the need for effective, ongoing monitoring and appropriate adaptive management strategies.

Although the population model has a built-in carrying capacity, and density dependence relationships that move the population towards this level, the effects of the environmental covariates are added independently of the density dependent mechanism, effectively changing the carrying capacity as a function of mean climatic conditions. This seems a realistic assumption as density dependence is likely to be the result of environmental factors which influence food availability and nesting success as well as of unrelated factors such as space limitations for nesting sites. The carrying capacity used here (of 12 000 breeding pairs) does not represent full use of available nesting sites. Based on the size of the island and nesting density, some 23 000 pairs could potentially nest on the island [14].

Forecast changes in maximum temperatures in the region of Albatross Island during the chick rearing season are predicted to reduce the breeding success and consequently the size of the shy albatross population compared with the climate neutral projection. A slight reduction in forecast rainfall does little to offset this. The A2 scenario predicts greater reduction than the B1 scenario. These population size losses could be offset, to an unknown degree, by increases in upwelling and consequent likely increases in prey availability. Further research forecasting this oceanographic phenomenon and its linkages with prey availability would be valuable. If incidental bycatch across all fishing operations were reduced by less than 50%, we predict that these reductions will not be completely offset (Fig 9). For the climate functional form used here (*b* = 1), population sizes were double (B1 scenario) or quadruple (A2 scenario) those forecast in the absence of any bycatch mitigation but remained below those of the climate neutral scenario in which no mitigation was implemented. Until the effect of increased upwelling is better understood, there is a need to consider further adaptation strategies such as drainage around nesting sites, shading of nests, supplementary feeding at colony sites and reduction in the spread of avian pox [58,59]. These adaptation strategies may offset the impacts of changes in climate.

Mitigation of seabird bycatch in longline fisheries has been an issue of conservation concern for some time [9,33]. More recently, attention has been focused on incidental bycatch in trawl fisheries [13,57,60,61]. Longline fisheries have a range of case studies to draw on when selecting mitigation devices that are both effective and operationally practical. Some of this experience is transferable to trawl operations. All longline vessels in Australia come under the Threat Abatement Plan (TAP, [62]) which requires that line vessels adopt mitigation measures. It sets in place bycatch trigger levels to ensure that action is taken when rates become excessive; its objective is to reduce the seabird bycatch rate in all areas, and seasons to below 0.05 birds per 1000 hooks for pelagic and 0.01 for demersal and new fisheries [62]. In 2011 (note that our model ends with 2010) AFMA introduced seabird management plans (SMPs) for all trawl vessels under their authority, and increased on-board monitoring of incidental bycatch on trawl vessels to reduce bycatch in the trawl sector of the SESSF (Mike Gerner, AFMA, pers comm). As such, bycatch is expected to reduce in the near future. Unfortunately studies into the levels of seabird bycatch prior to the adoption of these measures are not extensive [40] so that quantifying the effectiveness of recent changes relative to past practices will be problematic.

The identification of relationships between environmental factors and specific life history parameters or the spatial distribution of animal species is a first step in guiding effective seabird conservation in the face of climate variability. Enhanced understanding is achieved, where sufficient data are available, by models such as ours that synthesize long-term population studies, comprehensive life history characteristics, and that make use of the natural experiments that arise from past environmental fluctuation [58,63]. Such models are best placed to guide the selection of appropriate climate adaptation responses. Ideally, the next step would be to introduce aspects of the food web and predator-prey relationships into our models [58,63]. Another advance would be the integration of the processes of the mark-recapture models into the integrated framework [64] so that instead of conditioning the model on estimated survival and breeding success probabilities, the original banding data can be used and all the error associated with those (such as the risk of not observing a bird even when it has returned to breed) would be retained and translated into the future predictions. This would be particularly desirable in this shy albatross study because of difficulties with adult survival estimates

The effect of pulse perturbations on population demographics, in particular, the impacts of disease (avian pox) was not considered in this study. We removed two unexpectedly low breeding success rates that are likely to have been largely driven by disease prevalence (which is primarily evident during the stage immediately prior to fledging when large numbers of dying and dead chicks are observed in the colony). The mechanisms affecting pox outbreaks on Albatross Island are poorly understood but likely to be multi-factorial and complex [65] and although some environmental link is likely, are beyond the scope of the current project. However, such extreme events do influence population size and are likely to become more frequent under increased climate variability [66]. Pox years could be incorporated into the model as offsets that occur sporadically and could thus be propagated into the future. This could be done by linking the probability that an offset would occur in any given year with an environmental time series e.g. [67].

We did not evaluate the impacts of the environment on all possible demographic elements however, breeding success is considered to have the strongest response to environmental conditions [6,68]. Future work could include relationships with adult and juvenile survival rates, at-sea distributions, timing of breeding, recruitment to the breeding colony, and the breeding success of inexperienced birds relative to those of experienced birds, all of which have been shown to respond to environmental variability in some seabirds [22,69,70].

By simultaneously estimating the effects of fishing and the relationships between climate variables and breeding success within an integrated modeling framework that includes a population model, we were able to partition observed variability amongst these major influences. Moreover, we projected the population into the future using the outputs from climate forecast models to show that mitigation by fisheries must achieve at least a 50% reduction in bycatch rate in order to offset losses due to predicted future rainfall and temperature. Such an integrated modeling approach is recommended for future investigations of the effects of climate change on vulnerable populations, where multiple stressors exist.

## Supporting Information

S1 Appendix Detailed description of fishing fleets. (DOCX)

S2 Appendix Details on Albatross Island monitoring, survival rate estimation, and exploration of environmental co-variates. (DOCX)

S3 Appendix Technical description of albatross population model. (DOCX)
