# Supplementary material for: Effects of Climate Change and Fisheries Bycatch on Shy Albatross (Thalassarche cauta) in Southern Australia
Source: PLoS One. 2015 Jun 9;10(6):e0127006. doi: 10.1371/journal.pone.0127006 (PMC4461252; doi:10.1371/journal.pone.0127006)
Supplement: S1 Appendix — (DOCX) [file pone.0127006.s001.docx]

# S1 Appendix: Detailed description of fishing fleets

## Trawl data

The first diesel powered trawl vessels entered the South East Fishery (SET) in 1971 but were confined to New South Wales (NSW) ports until 1974 [1], thus the bulk of trawling effort is likely to have been outside of the shy albatross distribution until 1975. Record keeping commenced in earnest in 1978 with the Great Australian Bight Fishery (Nov 1977 to present) and the DPI, Victoria held database (1978 calendar year to present) so that only three years (1975-1977) of effort data are missing from the records, most of which is thought to have been concentrated in NSW [1]. Victorian and NSW-based fishing was brought under a Commonwealth management umbrella, reflected by the storage of the bulk of these data in the South East Trawl Fishery (August 1985 to present) database although Victorian effort was housed, for a time, in the Victorian Inshore Trawl Fishery database (May 2000 to 2007). Some inshore fishing by Victorian licensed vessels is still stored by DPI. Much of this fishing took place in the large bays and inlets of Victoria (e.g. Port Phillip Bay, Western Port bay, Corner Inlet). Although shy albatross are known to enter these large embayments they are not key foraging areas and so fishing in these locations was excluded. While the Commonwealth databases provide the exact location of fishing, locations in the DPI database are identified by blocks typically 15 minutes (approximately 21 x 28 km) in size. However, 10% of the otter trawl records were noted only as New South Wales waters, South Australian waters, or Tasmanian waters. These records were allocated to one of three 1˚ blocks just outside of the Victorian sector.

Midwater trawling for small pelagic fish began in 2000 (AFMA unpublished data). Associated effort data are stored in the Jack Mackerel Fishery (2000 to 2002) database, which became the Small Pelagic Fishery in 2002 (Dec 2002 to 2010). The Science Research Data extracted from CSIRO Marine and Atmospheric Research (CMAR) Data Warehouse database and the CSIRO data present in AFMA historical databases record a relatively large number of tows and so were included.

## Pelagic longline data

Pelagic (tuna and billfish directed) longline effort is recorded under the names AFMA, AFZIS Radio Reporting database (1980 to 1999), Tuna Fishery (1979 to 1999), Southern and Western Tuna and Billfish Fishery (SWTBF, 2000 to 2006), Eastern Tuna and Billfish Fishery (ETBF, 2000 to the present), Western Tuna and Billfish Fishery (WTBF, 2007 to the present). We assumed that the Radio Reporting and Tuna Fishery databases recorded all tuna fishing effort up to and including 1999/2000, after which data are captured by the SWTBF and ETBF databases.

## Demersal longline data

While demersal effort data were used in this work, these data were not likely to be influential as it is believed that far fewer seabirds are caught on demersal as compared with pelagic longlines [2]. Missing shark directed longline effort prior to the 1980s [3] is therefore not likely to influence results. The databases are the Southern Shark Hook fishery (Aug 1988 to August 1999), Southern Shark fishery (Aug 1989 to Dec 2002), South East Non-Trawl fishery (1997-2002), Gillnet, Hook and Trap fishery (2003-present).

## References

1. Tilzey RDJ. *The South East Fishery*. Bureau of Resource Sciences. Parkes, ACT; 1994.
2. Bugoni L, Neves TS, Leite NO Jr, Carvalho D, Sales G, Furness RW, et al. Potential bycatch of seabirds and turtles in hook-and-line fisheries of the Itaipava Fleet, Brazil. Fish Res. 2008;90: 217-224.
3. Walker T. *Galeorhinus galeus* fisheries of the world. In Shotton, R. (Ed). *Case studies* of the management of elasmobranch fisheries. FAO Fisheries Technical paper 378/2; 1999.
