# Supplementary material for: Effects of Climate Change and Fisheries Bycatch on Shy Albatross (Thalassarche cauta) in Southern Australia
Source: PLoS One. 2015 Jun 9;10(6):e0127006. doi: 10.1371/journal.pone.0127006 (PMC4461252; doi:10.1371/journal.pone.0127006)
Supplement: S2 Appendix — (DOCX) [file pone.0127006.s002.docx]

# S2 Appendix: Details on Albatross Island monitoring, survival rate estimation, and exploration of environmental co-variates

The shy albatross model is fitted to estimates of “observed survival rates” for adult and juvenile birds that were obtained from [14]. Additional information on the long running monitoring program that provided the mark-recapture information underlying those estimates (or “observations”) are given in the PhD thesis [20] of the first author of [14]. Relevant background information on the calculation of survival rates, and sampling intensity on Albatross Island, are repeated in this Appendix.

The relationship between breeding success of Albatross Island shy albatross and a large number of potentially influential environmental factors was investigated by [20], who identified significant relationships with local rainfall, air temperature, and sea-surface height (an indicator of upwelling) during the vulnerable chick-rearing stage. Relevant details of the investigation of environmental factors by [20] are repeated in this appendix.

All information provided in this appendix is sourced from [14] (sections 1 and 2) and [20] (section 3). The references cited therein are not repeated here. For additional information and original citations, see [14] and [20].

# Albatross Island monitoring

The shy albatross breeds on only three islands off the Australian coast: Albatross Island off the north coast of Tasmania, and Pedra Branca and Mewstone to the south. Four distinct shy albatross colonies exist on Albatross Island: North, South, Main and West. Very little population data is available prior to 1980, after which an annual monitoring program began. Monitoring consists of 1) counts of eggs; 2) aerial photographs of nesting sites; 3) calculation of nesting density multiplied by area occupied; 4) estimation of breeding success. Breeding success is defined as the proportion of eggs (counted at the end of the laying period in late September or early October) that successfully produce a chick (counted at the end of the breeding season just before fledging in late march/early April).

A count is made of the number of eggs laid. Subsampling is done for the larger Main and North colonies, which comprise 60% and 25% of the island’s breeding population; but all eggs are counted for the smaller South (12%) and West (3%) colonies.

Mark-recapture work is concentrated on the South colony where all birds have been banded as chicks since 1981. Only those birds whose ages are known through this means, are included in mark-recapture studies. In 2009 a total of 3983 chicks had been banded over 27 cohorts. Resightings of banded birds has been conducted annually since 1981. Greatest effort is directed at resighting breeding birds, with a total of 4536 resightings by 2009. Inconsistent effort has been directed towards resighting non-breeding birds, therefore these data were not used.

# Multi-state mark-recapture models

Multi-state mark-recapture models were used to estimate recruitment rates, and adult and juvenile survival. The models estimate 1) survival probabilities, 2) transition probabilities between states, and recapture probabilities. All models were fitted using M-Surge and model selection was performed using the quasi-Akaike Information Criterion (QAIC) which corrects for over-dispersion.

At the time of banding, individuals are in state 1 (juveniles). The resighting probability for state 1 is held fixed at zero because juveniles do not return to the colony after fledging until they are ready to breed (state 2 or 3). Heterogeneity in resighting probability noted, and model fits were improved by the use of two breeding states: state 2 for “general recovery” and state 3 for a more concentrated “study” having higher resighting probability. Transition probabilities between the two adult breeding states were held constant.

## Survival estimates

Because juvenile birds (state 1) are not resighted until they breed (state 2 or 3) it is not possible to estimate annual survival, only cumulative survival to age at first breeding. To simplify model parameterization, all juvenile mortality was assumed to occur during the first year at sea, and thereafter juveniles assumed the annual adult survival rates. Alternative parameterizations were considered where both adult and juvenile survival rates were assumed to be constant over time, or to vary annually.

The first step in modelling mark-recapture data is to find a model that gives an adequate fit to the data, to which subsequent models can be compared. The Jolly-Move model (JMV) and Conditional Arnason-Schwarz (CAS) models were both fitted and the resulting likelihood ratio test (F_35,255_) gave a chi-squared value similar to that obtained through the goodness of fit (GOF) test in U-Care: (80.462/35)/(553.660/255) = 1.0588. The likelihood ratio was not significant, indicating that both models fit equally well. It was therefore possible to use a variance inflation factor based on all components ($\hat{c}$ = 2.1866). The corresponding ‘umbrella’ model (in which transitions are age, state and time dependent) had a QAIC = DEV/2.1866 + 2 * np equal to 8011.629, a value that served as a benchmark for further model selection. As the starting model fitted the data with some over-dispersion, all subsequently preferred models could be assumed to fit the data.

A range of alternative models were trialled (Table S3.1). The smallest QAIC was given by the assumption of constant adult survival over all time and states (at 96.1% SE 0.45), but time varying survival for juveniles (Fig. S3.1). Recapture probability for breeding birds varied with time and state.

Table S3.1. Number of parameters, and fit criteria (Deviance, QAIC) for sixteen alternative models that were fitted by [14] to mark-recapture data. Survival for juveniles (applied to only the first year of life) and adults can be time varying “Time”, constant “Constant”, or incorporate a trend “Time trend”. The maximum age at which recruitment to the breeding colony occurred was fixed. The probability of recapture can vary by state, time or partial age and may or may not be separable (“State*Time” vs “State+Time”. (Adapted from Table 1, [14])

| Model | No. parameters | Deviance | QAIC | Juvenile Survival | Adult survival | Max recruitment age | Probability of recapture |
| --- | --- | --- | --- | --- | --- | --- | --- |
| 1 | 83 | 16926.66 | 7907.087 | Time | Constant | 9 | State*Time |
| 2 | 85 | 16921.49 | 7908.723 | Time | Constant | 9 | Time*State*Partial age |
| 3 | 85 | 16925.33 | 7910.477 | Time | Constant | 10 | State*Time |
| 4 | 104 | 16891.47 | 7932.995 | Time | Time | 9 | State*Time |
| 5 | 81 | 16997.98 | 7935.702 | Time | Constant | 8 | State*Time |
| 6 | 63 | 17081.6 | 7937.945 | Time | Trend | 9 | State*Time |
| 7 | 62 | 17105.8 | 7947.015 | Constant | Constant | 9 | State*Time |
| 8 | 87 | 17039.28 | 7966.589 | Constant | Time | 9 | State*Time |
| 9 | 83 | 17073.27 | 7974.138 | Constant | Time | 7 | State*Time |
| 10 | 61 | 17672.73 | 8204.286 | Constant | Time | 7 | State+Time |
| 11 | 39 | 18313.12 | 8453.158 | Constant | Constant | 9 | State+Time |
| 12 | 35 | 18336.61 | 8455.899 | Constant | Constant | 7 | State+Time |
| 13 | 33 | 18418.23 | 8489.227 | Constant | Constant | 6 | State+Time |
| 14 | 38 | 18800.37 | 8673.991 | Constant | Constant | 9 | Time |
| 15 | 10 | 19886.48 | 9114.705 | Constant | Constant | 6 | State |
| 16 | 8 | 20143.16 | 9228.092 | Constant | Constant | 5 | State |


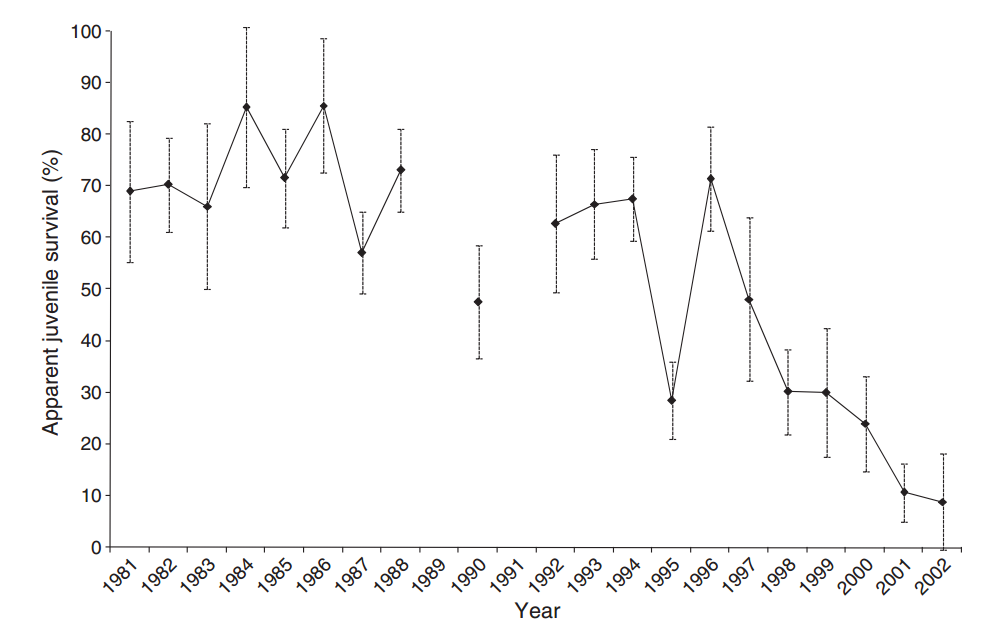


Figure S3.1. Juvenile survival rates for Albatross Island estimated from mark-recapture data by [14]. The apparent reduction in survival after 2003 is due to incomplete recruitment of younger cohorts. Error bars indicate standard error.

# Environmental factors

The absence of marked predation or disturbance of chicks on Albatross Island improves the likelihood of detecting relationships with environmental factors. The shy albatross breeding season was separated into three distinct life history phases “W” the winter pre-breeding period (May to August) 2), “I” incubation (September to December) and “C” chick-rearing (January to April). Shy albatross spend time on land at the breeding colony, and foraging at sea therefore both oceanic and land-based environmental variables were examined. Three core foraging areas were identified using satellite tracks for adult birds (5 collected during winter, 16 during incubation, and 7 during chick-rearing).

The oceanic environmental factors considered in each of these three (overlapping) areas were sea surface temperature (SST), chlorophyll concentration (CHLA) (a measure of ocean productivity), zonal westerly winds (ZWW) (a primary driver of water movement in the region), sea surface frontal density (SFD), and sea surface height anomaly (SSH) (an indicator of eddies, upwelling and down-welling).

The land based environmental factors were monthly mean maximum temperature and total rainfall (Rain) recorded at the nearest weather station (Cape Grim, 35km south east of Albatross Island). Maximum temperatures (MaxT) were examined because overheating can kill chicks. Breeding success increases with nest quality and marked rainfall degrades the nests (which are constructed from soil).

Additionally, the effect of the El Nĩno Southern Oscillation was considered using the Southern Oscillation Index (SOI). Monthly averaged SOI with lags of up to 24 months were considered, and the lag that gave the strongest uni-variate relationship with breeding success (5 months) was used in multi-variate analyses. None of these, including the 5 month lag, was significant.

## GLM

Environmental factors are unlikely to affect breeding success independently of one another, therefore multivariate analyses using generalized linear models (GLM) were applied. Due to over-dispersion in the breeding success data, a quasi-binomial distribution was assumed. Autocorrelation was not detected in the observed breeding success time series.

Co-linearity amongst environmental factors was investigated using the “corif” function in the R package “AED”. Using a cut-off value of 10, no variables were eliminated. GLMs were applied separately to data for each of the three phases of the breeding season (W, I and C). Maximum temperature was not considered during the winter pre-breeding period. Information theoretic rather than stepwise elimination was used to identify the minimally adequate model. The data were over-dispersed, so a QAIC statistic was used for model selection.

Coefficients, and fit criteria for the top ten ranked models for each of the three breeding phases are shown in Table S3.2, along with p-values for the top ranked models. On the basis of these results, six variables were chosen for the :global” model that would include SOI. These were ZWW.W, SDF.I, SSH.I, MaxT.C, Rain/C, and SSH.C. SSH during incubation (SSH.I) and chick rearing (SSH.C) were strongly correlated therefore a single, average, measure SSH.IC was generated. SOI, with a 5 month lag, was included in the global model.

The global model that has the lowest QAIC included only maximum temperature during the chick raising period (MaxT.C), rainfall during the same period (Rain.C) and sea surface height anomaly averaged of the chick rearing and incubation phases (SSH.IC), Table S3.2.

Table S3.2. Estimated coefficients for environmental factors included in GLM models for each of three breeding phases (W, I, C). Only the top ten ranked models are shown for each breeding phase, along with fit criteria (Deviance, QAIC and difference in QAIC compared with top ranked model). A blank indicates that an environmental factor was not included in the GLM. P values are shown for the top ranked models.

| Rank | MaxT* | Rain | Chla | SST | ZWW | SFD | SSH | Deviance | QAIC | ΔQAIC | Weight |
| --- | --- | --- | --- | --- | --- | --- | --- | --- | --- | --- | --- |
| W: Winter pre-breeding (May-Aug) | | | | | | | | | | | |
| *p* |  |  |  |  | *0.039* |  |  |  |  |  |  |
| 1 |  |  |  |  | 0.16 |  |  | 952.62 | 13.99 | 0 | 0.1 |
| 2 |  |  |  | 0.15 |  |  | -0.16 | 859.52 | 15.21 | 1.22 | 0.06 |
| 3 |  |  |  |  | 0.14 |  | -0.16 | 876.37 | 15.35 | 1.36 | 0.05 |
| 4 |  |  | -0.05 |  | 0.17 |  |  | 900.84 | 15.56 | 1.57 | 0.05 |
| 5 |  | -0.05 |  |  | 0.15 |  |  | 905.26 | 15.6 | 1.6 | 0.05 |
| 6 |  |  |  |  | 0.17 | -0.05 |  | 910.12 | 15.64 | 1.64 | 0.04 |
| 7 |  |  |  | 0.01 | 0.15 |  |  | 951.13 | 15.98 | 1.99 | 0.04 |
| 8 |  |  |  |  |  |  |  | 1431.12 | 16.01 | 2.02 | 0.04 |
| 9 |  | -0.14 |  | 0.15 |  |  |  | 954.89 | 16.01 | 2.02 | 0.04 |
| 10 |  |  |  |  |  |  | -0.10 | 1239.99 | 16.41 | 2.41 | 0.03 |
| I: Incubation (Sep-Dec) | | | | | | | | | | | |
| *p* |  |  |  |  |  | *0.051* | *0.006* |  |  |  |  |
| 1 |  |  |  |  |  | -0.14 | -0.21 | 620.46 | 16.04 | 0 | 0.09 |
| 2 |  | 0.10 |  |  |  | -0.14 | -0.26 | 480.46 | 16.23 | 0.19 | 0.08 |
| 3 | 0.07 | 0.12 |  |  |  | -0.13 | -0.30 | 418.39 | 17.43 | 1.38 | 0.05 |
| 4 |  |  |  |  | 0.05 | -0.12 | -0.21 | 574.84 | 17.45 | 1.41 | 0.04 |
| 5 |  | 0.10 |  |  | 0.05 | -0.13 | -0.26 | 440.93 | 17.72 | 1.67 | 0.04 |
| 6 |  | 0.11 | -0.05 |  |  | -0.16 | -0.27 | 443.69 | 17.75 | 1.71 | 0.04 |
| 7 | 0.03 |  |  |  |  | -0.13 | -0.23 | 602.73 | 17.81 | 1.77 | 0.04 |
| 8 |  |  | -0.03 |  |  | -0.15 | -0.22 | 605.74 | 17.85 | 1.81 | 0.04 |
| 9 |  |  |  | 0.01 |  | -0.13 | -0.21 | 617.93 | 18.01 | 1.97 | 0.03 |
| 10 |  |  |  |  |  |  | -0.16 | 927.75 | 18.03 | 1.98 | 0.03 |

Table S3.2. Continued from previous page.

| Rank | MaxT* | Rain | Chla | SST | ZWW | SFD | SSH | Deviance | QAIC | ΔQAIC | Weight |
| --- | --- | --- | --- | --- | --- | --- | --- | --- | --- | --- | --- |
| C: Chick-rearing (Jan-Apr) | | | | | | | | | | | |
| 1 | -0.13 | -0.13 |  |  |  |  | -0.11 | 298.92 | 16.49 | 0 | 0.11 |
| 2 | -0.18 | -0.21 |  |  |  | 0.09 |  | 356.75 | 17.75 | 1.26 | 0.06 |
| 3 | -0.15 | -0.16 |  |  |  | 0.05 | -0.09 | 265.46 | 17.77 | 1.27 | 0.06 |
| 4 | -0.2 | -0.17 |  | 0.09 |  |  | -0.12 | 273.2 | 17.93 | 1.44 | 0.05 |
| 5 | -0.14 | -0.19 |  |  |  |  |  | 465.89 | 18.12 | 1.63 | 0.05 |
| 6 | -0.13 | -0.13 |  |  | -0.02 |  | -0.12 | 293.34 | 18.37 | 1.88 | 0.04 |
| 7 | -0.13 | -0.13 | -0.01 |  |  |  | -0.11 | 297.5 | 18.46 | 1.97 | 0.04 |
| 8 | -0.17 | -0.21 | -0.05 |  |  | 0.09 |  | 312.97 | 18.8 | 2.31 | 0.04 |
| 9 |  |  |  | -0.13 |  |  | -0.13 | 509.18 | 19.06 | 2.57 | 0.03 |
| 10 | -0.16 | -0.17 |  |  | -0.04 | 0.07 | -0.1 | 246.88 | 19.36 | 2.87 | 0.03 |

| Rank | MaxT.C | Rain.C | ZWW.W | SFD.I | SSH.IC | SOI | Deviance | QAIC | ΔQAIC | Weight |
| --- | --- | --- | --- | --- | --- | --- | --- | --- | --- | --- |
| *p* | *0.010* | *0.044* |  |  | *0.058* |  |  |  |  |  |
| 1 | -0.14 | -0.12 |  |  | -0.11 |  | 306.42 | 20.92 | 0 | 0.16 |
| 2 | -0.19 | -0.15 | -0.09 |  | -0.14 |  | 251.12 | 20.95 | 0.03 | 0.16 |
| 3 | -0.17 | -0.15 | -0.12 | -0.07 | -0.17 |  | 195.15 | 20.96 | 0.03 | 0.16 |
| 4 | -023 | -0.17 | -0.13 |  | -0.18 | 0.06 | 216.29 | 21.71 | 0.79 | 0.11 |
| 5 | -0.11 | -0.12 |  | -0.04 | -0.12 |  | 279.37 | 21.96 | 1.04 | 0.1 |
| 6 | -0.21 | -0.17 | -0.15 | -0.06 | -0.20 | 0.05 | 167.92 | 21.99 | 1.06 | 0.1 |
| 7 | -0.14 | -0.12 | -0.01 |  | -0.12 | 0.01 | 303.73 | 22.83 | 1.9 | 0.06 |
| 8 | -0.12 | -0.12 | -0.05 | -0.04 | -0.13 | 0.01 | 279.05 | 23.95 | 3.03 | 0.04 |
| 9 | -0.14 | -0.19 |  |  |  |  | 465.89 | 24.61 | 3.68 | 0.03 |
| 10 | -0.13 |  |  |  | -0.18 |  | 492.87 | 25.57 | 4.65 | 0.02 |

Table S3.2. Estimated coefficients for environmental factors included in GLM models for each of three breeding phases (W, I, C). Only the top ten ranked models are shown for each breeding phase, along with fit criteria (Deviance, QAIC and difference in QAIC compared with top ranked model). A blank indicates that an environmental factor was not included in the GLM. P values are shown for the top ranked models.
