# Supplementary material for: Effects of Climate Change and Fisheries Bycatch on Shy Albatross (Thalassarche cauta) in Southern Australia
Source: PLoS One. 2015 Jun 9;10(6):e0127006. doi: 10.1371/journal.pone.0127006 (PMC4461252; doi:10.1371/journal.pone.0127006)
Supplement: S3 Appendix — (DOCX) [file pone.0127006.s003.docx]

# S3 Appendix: Technical description of albatross population model

The model used here is based on one originally presented by [11] and [50] for wandering albatross, but has been considerably altered. In particular:

- environmental variables are allowed to influence chick mortality and thereby, breeding success,
- estimation is achieved using likelihood instead of least squares,
- a monthly instead of annual time step is used,
- the model has been adapted to allow for breeding seasons of less than one full year (non biennial breeders),
- observed bycatch rates are now included along with other variables on which the model is conditioned,
- males and females are modelled separately,
- the population is not considered to be pristine at the start of significant fishing operations (due to egg and feather harvesting during the first half of the 19^th^ century).

# Within year dynamics

The death of either parent leads to breeding failure (the death of the chick) so that both male and female birds must to be modelled. For shy albatross, no differences have been observed between at-sea distributions for the two sexes, but the probability that a male is at sea, vulnerable to incidental capture, during the first month of the breeding season, is much lower than that of a female.

The model year begins on 1 October (model month $m$=1) when breeding commences. The number of birds hatched at the beginning of model year $y$ (designated as ‘chicks’) is given by $N_{y}^{0}$. From their second year of life until they first recruit to the breeding colony (given by an ogive) birds are ‘juveniles’. The population is divided into chicks, and an additional four categories (breeding $bx$, failed breeders $bf$, non-breeding$ns$, and juveniles $J$), so that the number of birds of sex *g*, and age $a$ and in any category $c$ during year $y$ and month $m$ is given by $N_{g,a,y,m}^{c}$,

$$N_{g,a,y,m}^{c}=\left[N_{g,a,y,m}^{bx},N_{g,a,y,m}^{bf},N_{g,a,y,m}^{ns},N_{g,a,y,m}^{J}\right].$$

Note that no distinction is made between non-breeding birds that were successful or unsuccessful in their previous breeding attempt as return rates for such birds have not been observed to differ for the Albatross island shy population (Rachael Alderman, unpublished data). Similarly, the likelihood of a bird attempting to breed during a particular year, is not thought to be affected by whether or not it made a breeding attempt in the previous year. At-sea distributions for this geographically restricted population differ little throughout the year so that the same adult and juvenile distributions are used both within and outside of the breeding season.

The model ‘year’ begins when birds arrive at their breeding colony (assumed to be the same day, for all birds of a given population) and ends at the end of February the following calendar year. On arrival, birds are assigned to the ‘breeding’ category $N_{g,a,y,m}^{bx}$ and, unless their breeding attempt fails during any month, they remain in this category until the end of the breeding season (at the end of month *Q*). Each month $m$, some surviving birds fail in their breeding attempt because their mate dies of natural causes (at instantaneous monthly rate $M/12$) or is caught by a fishery (at instantaneous monthly rate $F_{g,y,m}^{bx}$ for adult birds in category *c=bx*) or their chick dies (at instantaneous annual rate $M_{y}^{0}$/12). The number of surviving birds remaining in the breeding category at the start of month *m* is given by

$N_{g,a,y,m}^{bx}=N_{g,a,y,m-1}^{bx} e^{-(M/12+F_{g',y,m-1}^{bx}+M_{y}^{0}/12)} e^{-(M/12+F_{g,y,m-1}^{bx})} m=2\ldots Q$,

where $g'$is the sex of the mate of a bird of sex $g$.

Chick mortality $M_{y}^{0}$ and adult mortality $M$ are independent of sex and adult category, but fishing mortality $F_{g,y,m}^{c}$, because it depends on spatial overlap with fishing fleets differs between sexes *g*, and between adult (categories *bx, bf* and *ns*), and juvenile birds (category *J*) and changes monthly as at-sea distributions for fishing fleets vary.

Any breeding bird that fails in its breeding attempt during month *m-1* is moved into the failed breeders category at the start of the following month $N_{g,a,y,m}^{bf}$. The number of birds in the failed breeder category at the start of month *m* is given by those that remain from the previous month (that survive both natural and fishing mortality), plus those (surviving) breeders whose breeding attempt failed during the previous month $m$*-1*,

$$N_{g,a,y,m}^{bf}=N_{g,a,y,m-1}^{bf} e^{-(M/12+F_{g,y,m-1}^{bx})}$$

$$+N_{g,a,y,m-1}^{bx} \left[1-e^{-(M/12+F_{g',y,m-1}^{bx}+M_{y}^{0} /12)}\right] e^{-(M/12+F_{g,y,m-1}^{c})} m=2\ldots Q$$

Once the breeding season is over, all surviving breeding or failed birds move into the non-breeding category alongside surviving birds that did not attempt to breed during year *y*,

$N_{g,a,y,Q+1}^{ns}=N_{g,a,y,Q}^{ns} e^{-(M/12+F_{g,y,Q}^{ns})}+N_{g,a,y,Q}^{bx} e^{-(M/12+F_{g,y,Q}^{bx})}+N_{g,a,y,Q}^{bf} e^{-(M/12+F_{g,y,Q}^{bf})}$ (1)

$N_{g,a,y,Q+1}^{bx}=N_{g,a,y,Q+1}^{bf}=0$.

Adult birds that did not attempt to breed during year $y$ remain in the non-breeding category for the duration of the year, and suffer natural $M$ and fishing mortality ($F_{g,y,m}^{ns}$) during each month (the number of non-breeding birds at the start of month *m=Q+1* is given by equation 1)

$N_{g,a,y,m}^{ns}=N_{g,a,y,m-1}^{ns} e^{-(M/12+F_{g,y,m-1}^{ns})} m=2\ldots(Q-1),(Q+1)...12$.

Similarly, juvenile birds remain in the juvenile category throughout the month and suffer both natural and fishing mortality. Juveniles have their own, sex (although these do not differ, for shy albatross), age and density dependent natural mortality rate $M_{a,y}^{J}$ and fishing mortality rate (see Section 3),

$N_{g,a,y,m}^{J}=N_{g,a,y,m-1}^{J} e^{-(M_{a,y}^{J}/12+F_{g,y,m-1}^{J})} m=2\ldots12.$ (2)

Any chicks that survive to fledge at the end of the breeding season, are added to the juvenile category, age 0, at the beginning of month *Q*+1. For clarity of presentation, this is not shown in equation 2.

All eggs are assumed to be laid on the first day of the breeding season. The number of chicks (including eggs) present in the colony during the breeding season at the start of month *m* is given by those that survive from the previous month. Death occurs due to natural causes (e.g. environmental, or physiological) $M_{y}^{0}$ or through the natural $M$ or fishing related $F_{g,y,m}^{bx}$ death of either parent,

$$N_{g,y,m}^{0}=N_{g,y,m-1}^{0} e^{-M_{y}^{0} /12-\sum_{g'=1}^{2} \left(M/12+F_{g',y,m-1}^{bx}\right)} m=2\ldots Q.$$

At the end of the breeding season all chicks move into the juvenile category and become vulnerable to incidental catch by fisheries (equation 2).

# Between year dynamics

At the end of each year (i.e. start of the next year), all birds move to the next age class, a proportion of each juvenile age group recruits into the breeding adult category, and some adult birds move from the non-breeding to the breeding category.

The number of breeding birds of sex *g* and age $a+1$ at the start of year $y+1$ (month $1$) $N_{g,a+1,y+1,1}^{bx}$ is given by the sum of the number of returning failed breeders (at rate $\varphi_{y}$), the returning non-breeders (also at rate $\varphi_{y}$), and the maturing juveniles (at age-specific rate $\lambda_{a}$),

$$N_{g,a+1,y+1,1}^{bx}=\varphi_{y}\left(N_{g,a,y,13}^{bf}+ N_{g,a,y,13}^{ns}\right) +\lambda_{a} N_{g,a,y,13}^{J} a=1...A-2$$

where *A* is the maximum age and is a (a plus group) so that,

$N_{g,A,y+1,1}^{bx}=N_{g,A,y,13}^{bx}+N_{g,A-1,y,13}^{bx}+\varphi_{y}\left(N_{g,A-1,y,13}^{bf}+ N_{g,A-1,y,13}^{ns}\right) +\lambda_{A-1} N_{g,A-1,y,13}^{J}$.

Note that ‘13’ indicates the number of birds present at the very end of month 12 of year $y$ (i.e. the start of ‘month 13’) and is used for clarity of presentation so that mortality tht occurred during the last month of the year need not be shown.

No birds are assigned to the failed breeder category at the start of the year,

$$N_{g,a+1,y+1,1}^{bf}=0 g=1,2; a=2...A-1.$$

Some adult birds take a year’s sabbatical (at rate $1-\varphi_{y}$),

$$N_{g,a+1,y+1,1}^{ns}=\left(1-\varphi_{y}\right) N_{g,a,y,13}^{ns} g=1,2; a=2...A-1.$$

Juvenile birds leave to join the adult breeding category at age-specific rate $\lambda_{a}$ (note that surviving chicks from the previous year *y* became juveniles of age zero $N_{0,y,Q+1}^{J}$ during that year, at the end of the breeding season),

$N_{g,a+1,y+1,1}^{J}=\left(1-\lambda_{a}\right) N_{g,a,y,13}^{J} a=0... A-1$.

The number of breeding pairs $B_{y,m}$ present in the colony during the first month of the breeding season is given by the smaller of the number of breeding males and breeding females. Shy albatross raise a single chick so the number of breeding pairs is also the number of chicks (or eggs) $N_{y,1}^{0}$ at the start of the breeding season (*m*=1). Assuming a 1:1 sex ratio at birth

$N_{g,y,1}^{0}=0.5 B_{y,1}=\min\left( \sum_{a=1}^{A} N_{1,a,y,1}^{bx}, \sum_{a=1}^{A} N_{2,a,y,1}^{bx}\right).$ (3)

# Density dependence on juvenile mortality

Density dependence for juveniles is modelled by, initially, making two key assumptions; first that the lowest mortality rate for juveniles (achieved at population size zero) is equal to that of adults $M$; and second, that juvenile mortality increases linearly with increasing population size, to its maximum rate $M_{0}^{J}$ when the population is at its unfished equilibrium size $N_{0}^{1+}$. The $1+$ population size is given by the total number of birds aged $a=1$ or more, in all categories $c$ in the population at the start of year $y$ (month $m=1$), $N_{y}^{1+}$,

$N_{y}^{1+}=\sum_{c} \sum_{g=1}^{2} \sum_{a=1}^{A} N_{g,a,y,1}^{c}$.

By simple linear regression (linear, that is, if $\gamma$=1), for any population size $N_{y}^{1+}$ the corresponding density dependent juvenile mortality rate $M_{y}^{J}$ is,

$M_{y}^{J}=M+\left(M-M_{0}^{J}\right) \left(\frac{N_{y}^{1+}}{N_{0}^{1+}}\right)^{\gamma}$.

Juvenile birds assume natural mortality rate $M_{y}^{J}$ to the age of 5, thereafter taking the adult natural mortality rate $M$. The shape of this function is governed by the parameter $\gamma$, which is estimated.

# Density dependence on chick mortality

Density dependence is modelled for the chick mortality term as a function of the number of breeding pairs in the population$B_{y}$. Chick mortality (before accounting for environmental effects $\bar{M}_{y}^{0}$) is assumed to be at its lowest rate at zero population size, when it is assumed to be equal to that of adults *M.* At maximum population size chick mortality is $M_{0}^{0}$

$\bar{M}_{y}^{0}=M \exp\left[\left(B_{y}/B_{0}\right)^{\kappa} \ln\left(M_{0}^{0}/M\right)\right]$ (4)

where

(5)**.**

The highest rate of chick mortality $M_{0}^{0}$ reflects the mortality of both parents during the portion of the year represented by the breeding season $p^{B}$, and the breeding success when the population is at unexploited equilibrium $\tilde{S}_{0}$, which is an estimated model parameter. Given a 5 month breeding season (1 Oct to end Feb) $p^{B}$=5/12. The parameter $\kappa$ controls the level of density dependent compensation ($\kappa$=0 gives density independent chick mortality) and is also an estimated parameter.

Environmental variables further influence the realized chick mortality rate in year *y* $M_{y}^{0}$, see section 5.

# Environmental variables

Environmental variables cause the chick mortality rate $M_{y}^{0}$ in a given year *y* to deviate from the average level $\bar{M}_{y}^{0}$ given by equation 4)

$$M_{y}^{0}=\bar{M}_{y}^{0} \sum_{i\in I} f\left(x_{i}\right)$$

Where $f\left(x_{i}\right)$ is a functional relationship for environmental covariate *x_i_* (of the set of covariates *I* used by the model). A flexible, exponential, functional form was used

$f\left(x_{i}\right)=\exp\left(\theta_{i} x_{i}^{b}\right)$.

Here the parameters $\theta_{i}$, termed “slope” parameters are estimated by the model whereas the *b* parameter is fixed (values of *b* = 0.5, 1 and 2 were explored).

# Modelling incidental catch

The instantaneous fishing mortality rate $F_{g,y,m}^{c}$ for birds in any category $c$ of sex *g* during month $m$ of year $y$ is calculated from the number of birds caught during the month $C_{g,y,m}^{c}$ by assuming that all birds are caught in a pulse at the middle of each month, after half the month’s natural mortality has occurred,

$$F_{g,y,m}^{c}=-log\left(1-\frac{C_{g,y,m}^{c}}{\sum_{a=1}^{A} N_{g,y,m}^{c} e^{-0.5 M/12}}\right)$$

Note that fishing mortality rates will be the same for all adult categories because at-sea distributions are the same but that juveniles have a different distribution and therefore different (likely greater due to their larger foraging range) exposure to fisheries mortality,

$F_{g,y,m}^{bx}=F_{g,y,m}^{bf}=F_{g,y,m}^{ns}\neq F_{g,y,m}^{J}$.

Juveniles may also be more susceptible to fishing than adults due to their inexperience [54]. In contrast however, juveniles may be competitively inferior to adults at longlines and when both are present adults may be caught on baited hooks more often than juveniles (J. Croxall, pers. comm.). [50] found there were insufficient data to estimate differential catchability between adults and juveniles, therefore, in the present study the catchabilities are assumed to be equal.

To model the total catch $C_{y,m}^{c}$ of birds from category $c$ of taken during month $m$ of year $y$, the catch of birds of both sexes *g* and all ages *a* in each 1˚ block *B* by each fishery $f$ is summed. The catch in a particular block *B* is a function of the number of birds in the population at the middle of the month $\overline{N}_{g,a,y,m}^{c}$, present in block *B* at that time. Presence in block *B* is given by $P_{y,m,B}^{c}$ the proportion of the birds from category $c$ that have been observed to occupy block *B* (i.e. the at-sea distribution), multiplied by $P_{g,m}^{S}$ the number of birds that are likely to be at sea. This is, in turn, multiplied by the effective number of hooks deployed in that block at that time, which is given by the product of the total recorded number of hooks $E_{y,m,B}^{f}$ deployed by each fishery $f$ in month *m* and block *B* multiplied by the model estimated ‘catchability’ of hooks for fishery $f$, $q^{f}$. The total effective number of hooks is calculated by summing across all fisheries $f$. The total catch during month $m$ of year $y$ is given by summing over all ages $a$ and 1˚ blocks *B*,

$C_{y,m}^{c}=\sum_{g,a,B} \left(\overline{N}_{g,a,y,m}^{c} P_{y,m,B}^{c} P_{g,m}^{S} \sum_{f} q^{f} E_{y,m,B}^{f}\right).$ (6)

The probability that a bird is at sea is a function of whether or not it is incubating an egg or chick and therefore differs by category *c* and month *m* as well as sex *g*. These values were taken from field observations (Rachael Alderman, unpublished data) and are set to 0.95 for females and 0.45 for males in September, and 0.5 for both sexes during October to December and to 1 for all birds at all other times of the year.

The number of birds in the middle of the month $\overline{N}_{g,a,y,m}^{c}$ is given by the product of the numbers at the start of the month $N_{g,a,y,m}^{c}$ and half the natural mortality for birds of age $a$ in category $c$.

# Matching observed bycatch rate

A single observed bycatch rate is available for the trawl super-fleet [43]. The model estimates a bycatch rate for the study area (matched as closely as possible by several 1˚ spatial blocks) over the period of the study by summing the number of birds caught $C_{y,m,B}^{c}$ over the relevant months and blocks of the study, and dividing trawl effort $E_{y,m,B}^{f}$ summed over the same time and space.

# Initial conditions

We calculate the juvenile natural mortality rate $M_{0}^{J}$ that would maintain the population at pristine equilibrium, given the resource dynamics equations shown in this appendix, the (model estimated) adult natural mortality rate $M$, the pristine chick natural mortality rate $M_{0}^{0}$ (from equation 5 and model estimated parameter $\tilde{S}_{0}$), and the number of breeding pairs present at pristine equilibrium $B_{0}$ (assumed to be 12 000 pairs).

The population is not assumed to be pristine at the start of the modelling period, although it is assumed to be at equilibrium. Incidental bycatch in fisheries is assumed not to have occurred, but a steady rate of anthropogenic monthly mortality *X* (attributed to egg and feather harvesting) is assumed to have been applied to all sexes *g*, ages *a* and categories *c*. Age structure at the start of the modelled period (*y* = *m* = 1) is the number of chicks present at the start of year *y*=1 (given by the number of breeding pairs $B_{1,1}$ and the breeding success $\tilde{S}_{1}$) and the annual mortality rates *M* and *X*,

$N_{g,a,1,1}^{c}=B_{1,1} \tilde{S}_{1} e^{-\left(a-1\right)(M+X)}$*a=1…A.* (7)

An iterative approach was used to estimate the size of the population that resulted from a mortality rate of *X*, given the density dependent compensation in juvenile and chick mortality rates corresponding to that population size.

The earliest population count deemed reliable enough to include in the model is an estimate of the number of breeding pairs nesting in 1972, long after albatross harvesting had ended [14]. Notable fisheries effort, and associated bycatch, commenced in the early 1980s. Therefore we start the modelled period 40 years prior to this, in 1942, with a population that is assumed to have suffered a steady rate of additional (harvest-related) mortality applied evenly to all age classes and evenly across years. The actual distribution of mortality across age groups is unknown, except that fledged juveniles would not have been harvested prior to their return to the colony for breeding. However, eggs were likely harvested as well as adults so that the impact would have been felt across the age range. Furthermore, the impact of harvesting on the age structure of the population would reflect changes in the intensity of harvesting over time. This, and even the exact date when harvesting ended (by at least the middle of the 19^th^ century), are unknown. We therefore commence the model at least one albatross lifetime prior to the collection of data (in 1942) in order to simulate a population that has experienced a long period of recovery. The residual effects on the age structure of the population due to harvesting that was mosl likely unevenly applied across cohorts and across lifetimes will have faded to insignificance over the years so that, by the time the model begins comparing the population with data, in 1972, the effect of the incorrect assumption of evenly applied harvesting, will be inconsequential.

# Response variables

The demographic response variables (i.e. for which data time series exist) are the numbers of breeding pairs at the start of each year $B_{y,1}$, the numbers of chicks fledged at the end of the breeding season $N_{0,Q+1}^{J}$, the annual adult survival rate $S_{y}^{A}$, and juvenile survival to age 5 $S_{y}^{J}$.

The number of breeding pairs in the population at the time of census $B_{y,1}$ is given by equation 3. The breeding success $\tilde{S}_{y}$ in year *y* is given by the number of chicks fledged at the end of the breeding season $N_{0,Q+1}^{J}$ (they are allocated to the juvenile category at the start of the first month after the breeding season ends, *Q*+1) divided by the number of pairs that made a breeding attempt,

$$\tilde{S}_{y}=N_{0,Q+1}^{J}/B_{y,1}$$

Adult survival $S_{y}^{A}$ (a percentage) is given by,

$$S_{y}^{A}=100 N_{y+1}^{A}/N_{y}^{A}$$

where

$N_{y}^{A}= \sum_{a=1}^{A} \sum_{c\in\left(bx,bf,ns\right)} N_{a,y,1}^{c}$.

and *A* is the maximum age group in the model (a plus group).

The juvenile survival rate to age 5 (a percentage) is,

$S_{y}^{J}=100 e^{-Z_{y}^{J}}$,

where $Z_{y}^{J}$ is the total mortality rate over the previous 4 years for juveniles that are aged 5 at the start of year $y$,

Juvenile survival to age 5 $S_{y}^{J}$ cannot easily be expressed in terms of a ratio of the numbers of juveniles present in various years because birds leave the juvenile category through maturation, not just through mortality.

Note that the annual breeding success is given by the number of chicks fledged divided by the number of breeding pairs. Therefore one can choose to condition the model on any two of the three quantities: number of breeding pairs, number of chicks fledged, or breeding success.

Finally, a response variable was calculated to match the observed bycatch rate, representing numbers of birds per thousand trawl hours, observed in a specified set of 1˚ blocks over a span of months by the trawl super-fleet. This was given by the estimated catch in number of birds $C_{i}$ divided by the effort in thousands of trawl hours $E_{i}$ over that region and time, for the single bycatch observation *i*,

$R_{i}=C_{i}/E_{i}$.

The number of birds caught $C_{i}$ is estimated in the same way as $C_{y,m}^{c}$ in equation 6, with appropriate summation over 1˚ degree blocks and time periods and over all categories $c$.

# Likelihood

Maximum likelihood was used to estimate the model parameters. Normal distributions are assumed for the residuals for the expected $B_{y,1}$ and observed $B_{y,1}^{obs}$ number of breeding pairs,

$B_{y,1}$ ~ $N \left(B_{y,1}^{obs}, \sigma_{B}\right)$

for all years *y* for which observations exist. Similarly, a normal distribution is assumed for the residuals of the expected $\tilde{S}_{y}$ and observed $\tilde{S}_{y}^{obs}$ breeding success for all years *y* for which observations exist,

$\tilde{S}_{y}$ ~ .

Values of $\sigma_{B}$ = 405 and $\sigma_{\tilde{S}}$ = 0.015 were obtained using iterative re-weighting.

A binomial distribution was assumed for the adult survival rate, along with an assumed effective sample size of 100 animals, which seemed to achieve a good balance with the other data sources. The binomial probability was given by the observed survival rates$p=S_{y,1}^{A,obs}$, and the number of observations was the integer part of ($100 S_{y}^{A}$) out of 100 trials.

$100 S_{y}^{A}$ ~

Similarly, for juvenile survival,

$100 S_{y}^{J}$ ~

A normal distribution was assumed for the single observed bycatch rate of 12.66 birds per thousand trawls. As iterative reweighting is not possible given a single value, a value of 1 was chosen, somewhat arbitrarily, to give a fit to the bycatch observation that did not dominate, or neglect, that data source.

# Estimated parameters and standard errors

The estimated parameters of the model are the breeding success at unexploited equilibrium $\tilde{S}_{0}$; the adult natural mortality rate *M*, the additional mortality prior to the start of modelling *X*; the parameters governing density dependence for juvenile birds *γ*, and for chicks κ; the catchability parameters for the pelagic line $q^{p}$, demersal line $q^{d}$ and trawl $q^{t}$ super-fleets; and the three environmental parameters $\theta_{i}$. The juvenile density dependence *γ* and demersal catchability $q^{d}$ parameters were estimated to be zero for all forms of the model explored.

Standard errors for parameter estimates were approximated using the Fisher information matrix derived from the Hessian evaluated at the maximum likelihood.
